# Understanding the Toxicity of Carbon Dots: The Role of Synthesis Variability, Surface Chemistry, and Biological Context

**DOI:** 10.3390/ijms27093782

**Published:** 2026-04-24

**Authors:** Hasan Shabbir, Yanwen Chen, Jing Sun, Magdalena Kotańska, Noemi Nicosia, Edit Csapó, Marek Wojnicki

**Affiliations:** 1 Faculty of Non–Ferrous Metals, AGH University of Krakow, Mickiewicza Ave., 30-059 Krakow, Poland; 2 Department of Gastroenterology, Ruijn Hospital, Shanghai Jiao Tong University School of Medicine, Shanghai 200025, China; 3 Department of Pharmacodynamics, Laboratory of Pharmacological Screening, Jagiellonian University Medical College, Medyczna 9, 30-688 Cracow, Poland; magda.dudek@uj.edu.pl; 4 PhD Program in Neuroscience, School of Medicine and Surgery, University of Milano-Bicocca, 20900 Monza, Italy; 5 School of Medicine and Surgery, University of Milano-Bicocca, 20900 Monza, Italy; 6 MTA-SZTE Lendület “Momentum” Noble Metal Nanostructures Research Group, University of Szeged, Rerrich B. Sqr. 1, H-6720 Szeged, Hungary; juhaszne@chem.u-szeged.hu; 7 Interdisciplinary Excellence Center, Department of Physical Chemistry and Materials Science, University of Szeged, Rerrich B. Sqr. 1, H-6720 Szeged, Hungary

**Keywords:** carbon quantum dots, cytotoxicity, biocompatibility, photoluminescence, synthesis methods, bioimaging, drug delivery, nanomaterials, antibacterial properties, in vivo toxicity, surface functionalization, reactive oxygen species, biomedical applications, toxicity evaluation

## Abstract

Since their initial discovery in 2003, carbon quantum dots (CDs) have attracted significant attention due to their unique optical properties and potential biomedical applications. This review critically examines the past 20 years of research on CDs, with a particular focus on cytotoxicity studies from the last decade. CDs, typically less than 10 nm in size, have been synthesized from various organic and inorganic precursors using multiple methods, including hydrothermal, microwave, and chemical reduction techniques. Their properties can be finely tuned by modifying synthesis parameters and incorporating dopants. The preliminary studies on the biological effects of CDs were published in 2013, highlighting their antibacterial properties and low toxicity in certain contexts. Subsequent research has explored their bioactivity, including their application in drug delivery, bioimaging, and photothermal therapy. However, the cytotoxicity of CDs remains a critical area of investigation. Further studies have demonstrated that surface functional groups, charge, concentration, and size significantly influence their interaction with biological systems. For instance, CDs with positive surface charges exhibit higher cellular uptake and greater cytotoxicity compared to their negatively charged counterparts. In vivo studies utilizing animal models such as zebrafish, mice, and planarians have provided valuable insights into the potential toxicological impacts of CDs. The results indicate that while CDs generally exhibit low toxicity at certain concentrations, high doses can lead to adverse effects, including oxidative stress, organ damage, and disrupted cellular functions. Notably, the route of administration (oral, intravenous, or intraperitoneal) also affects the observed toxicity profiles. The goal of this review is to integrate the results of various studies to provide a balanced perspective on the potential risks and benefits of CDs, guiding future research and applications in nanomedicine. This review underscores the necessity for standardized and comprehensive toxicological evaluations of CDs to fully understand their safety and efficacy for biomedical applications.

## 1. Introduction

Carbon dots (CDs) have emerged over the past two decades as a versatile class of carbon-based nanomaterials with broad applications in bioimaging, sensing, and drug delivery [1,2]. Their perceived advantages—including tunable photoluminescence and aqueous dispersibility—have led to a rapidly expanding body of literature [3]. However, conclusions regarding CD safety remain highly inconsistent, ranging from claims of excellent biocompatibility to reports of pronounced cytotoxicity. These discrepancies are rarely addressed systematically, making biological outcomes difficult to compare across studies.

An additional source of ambiguity arises from the frequent conflation of CDs with graphene quantum dots (GQDs). Although both are carbon-based, they differ fundamentally in dimensionality, structural order, and biological interactions. Treating them as interchangeable may obscure material-specific toxicity mechanisms.

**Figure 1:**
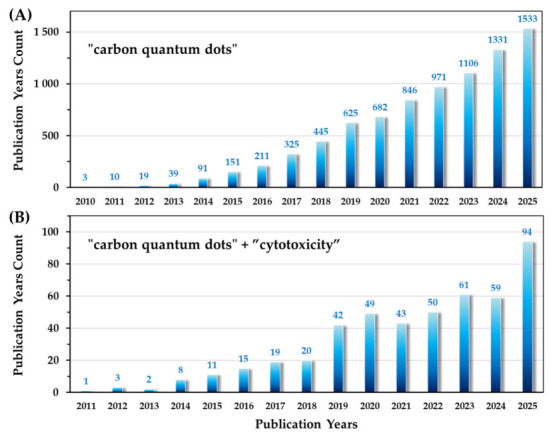
Number of published papers on carbon quantum dots (**A**) and on CD cytotoxicity (**B**) based on a literature survey performed using the database.

Figure 1A,B illustrates the temporal discrepancy between the rapid growth in CD synthesis and the delayed emergence of toxicity-oriented investigations. While interest is growing, the field remains characterized by a high density of data points but limited conceptual consolidation. This review covers studies primarily from the past decade, treating CDs as a distinct class and comparing them with GQDs where relevant.

Given that carbon dots represent a highly heterogeneous class of nanomaterials, this review aims to move beyond a descriptive compilation of reports toward a mechanistic analysis of CD biosafety. Rather than asking whether CDs are inherently “toxic,” we focus on identifying the key physicochemical and experimental parameters—such as surface functionalization, exposure conditions, and methodological limitations—that govern biological responses. By consolidating existing evidence and highlighting unresolved challenges, this review provides a conceptual framework for future safety assessments and reproducible research.

This review covers studies published primarily over the past decade, with selective inclusion of earlier foundational works where mechanistic insight is required. The following databases were reviewed, Web of Science, PubMed, and Scopus, based on the following keywords: “carbon quantum dots” and “cytotoxicity”.

## 2. Classification of Carbon-Based Quantum Dots

They also used the electron microscope at different stages of synthesis and investigated the growth of nuclei at each stage. When L-Cysteine, glucose, and citric acid were applied as carbon source and mixed with 2-aminoethanol (also known as monoethanolamine) at room temperature, nucleus formation was not observed by electron microscopy. The researchers studied the growth process of CDs as a function of the temperature, observing increased growth at higher temperature compared with room temperature. At 130 °C, large nanoparticles of approximately 150 nm were formed in the solution. Next, at 150 °C, the nanoparticles dehydrated and their size decreased to 60 nm. At 170 °C, a 1.5 nm CD polymer sheet was observed. When 170 °C, was maintained for 10 min, the polymer sheet disappeared and CDs with a size of 3.5 were observed.

Wenquan Shi et al. [4] investigated four different precursors that were used to synthesize CDs: carbon powder, carbon nanotubes, and graphite. They showed that the surface functionalization and properties of CDs are influenced by the synthesis method, while the core structure and chemical composition are influenced by the precursor.

### 2.1. Carbon Dots as a Broad and Heterogeneous Material Class

Carbon dots (CDs) represent a broad class of carbon-based nanomaterials typically characterized by quasi-spherical morphology, nanoscale dimensions (generally below 10 nm), and size- and surface-dependent photoluminescence. CDs are the most synthesized via bottom-up approaches, including hydrothermal, solvothermal, microwave-assisted, and pyrolytic methods, using a wide range of molecular or polymeric precursors. Consequently, these synthetic pathways result in CDs frequently displaying considerable structural and chemical variability, which includes a carbonaceous core, surface functional groups, molecular fluorophores, and differing levels of graphitic order.

This intrinsic heterogeneity has important implications for biological interactions and toxicity assessment. Unlike well-defined inorganic quantum dots, CDs do not represent a single, chemically uniform material. Instead, their physicochemical properties (including size distribution, surface charge, degree of oxidation, and chemical composition) are highly sensitive to precursor selection and synthesis conditions. As such, toxicity data obtained for one CD formulation cannot be readily extrapolated to others without careful consideration of these parameters.

### 2.2. Graphene Quantum Dots: Structurally Distinct Two-Dimensional Nanomaterials

Graphene quantum dots (GQDs) are frequently discussed alongside carbon dots due to shared carbon-based composition and quantum confinement-related optical properties. However, GQDs are fundamentally distinct materials, consisting of nanoscale fragments of graphene with a predominantly two-dimensional structure, extended sp^2^-conjugation, and well-defined graphitic domains.

The planar geometry of GQDs, combined with their high surface area and π–π stacking capability, leads to biological interactions that differ markedly from those of quasi-spherical CDs. GQDs often display stronger interactions with bio-membranes, nucleic acids, and aromatic biomolecules, as well as a higher propensity for aggregation under physiological conditions. These characteristics can influence cellular uptake pathways, intracellular localization, and toxicity mechanisms.

For the first time, Yu Chong et al. [5] performed systematic studies about graphene quantum dots, highlighting their difference compared to carbon quantum dots. The main differences between graphene quantum dots and carbon quantum dots lie in their size, morphology, and precursors used for their synthesis. CDs are typically spherical or semi-spherical carbon particles with sizes less than 10 nm. On the other hand, graphene quantum dots (GQDs) are two-dimensional materials consisting of one or a few layers of graphene, with sizes ranging from 2 to 20 nm. Additionally, the precursors used for synthesizing these materials differ significantly. GQDs are derived from graphene-based materials, while CDs can be synthesized from various compounds including bio-waste, plant extracts (green synthesis), and pure chemicals (gray synthesis). The authors performed in vivo studies using BALB/c mice. GQDs-PEG surface was modified with Cy7 to increase the detectability of GQDs obtaining particles with size ca. 2 nm. In addition, further modifications of GQDs using PEG1000 and Cy7, were essential to study the toxicity of the composite rather than GQDs. Therefore, these results should be considered preliminary, as the authors did not demonstrate that modification of GQDs using Cy7 is stable. In the conclusion, the authors suggest that the obtained results of WST-1 assay, cell apoptosis, LDH production, and ROS level clearly demonstrated good biocompatibility of GQD and GQD-PEG at the cellular level. NIR imaging of the mice administered with GQD-PEG suggested that GQD could be metabolized quickly through the kidneys and be retained in the tumor, regardless of administration routes.

### 2.3. Implications of CDs–GQDs Conflation for Toxicity Interpretation

Despite these fundamental differences, CDs and GQDs are frequently grouped together in toxicity studies and reviews, with the results discussed interchangeably or generalized across material classes. Such conflation can obscure material-specific trends and contribute to apparent inconsistencies in reported toxicity outcomes.

For example, observations related to membrane disruption, oxidative stress, or genotoxicity reported for GQDs may not be directly transferable to CDs with predominantly zero-dimensional morphology and distinct surface chemistry. Conversely, conclusions regarding the apparent biocompatibility of certain CDs may not apply to GQDs exhibiting stronger π–π interactions and higher structural order.

In this review, carbon dots and graphene quantum dots are therefore treated as related but distinct nanomaterial classes. Toxicity data for GQDs are discussed separately and comparatively where appropriate, with explicit attention to how differences in dimensionality, surface chemistry, and structural order influence biological responses.

### 2.4. Carbon Dots Derived from Natural Precursors: Implications for Biosafety

Sonam Mandani et al. showed that CDs can be obtained from pure natural materials without any processing, extracting CDs from natural honey. CDs have properties like those prepared by other top-down or bottom-up methods. The use of CDs by humans has been documented in the literature for centuries. Many papers report on the usage of raw honey from a beehive to extract CDs. According to the protocol, raw honey is dialyzed with water for 48 h to remove free amino acids, carbohydrates, ions, vitamins, etc., to obtain the final supernatant, yielding 15 mg CDs/gram of honey after lyophilization. The solution appears golden yellow in sunlight, whereas under UV light it emits blue fluorescence [6].

**Figure 2:**
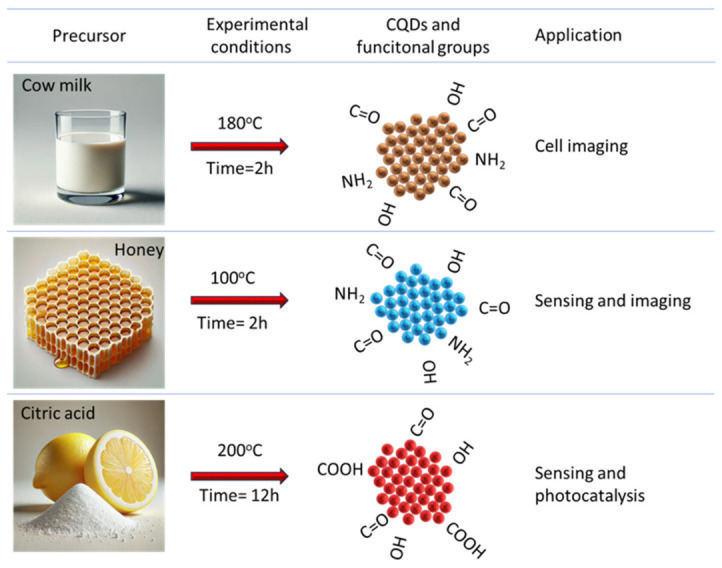
Schematic representation illustrates how the chemical structure of precursor materials governs heteroatom doping and the formation of surface functional groups in carbon quantum dots (CQDs), ultimately shaping their physicochemical properties and potential biological responses.

**Figure 3:**
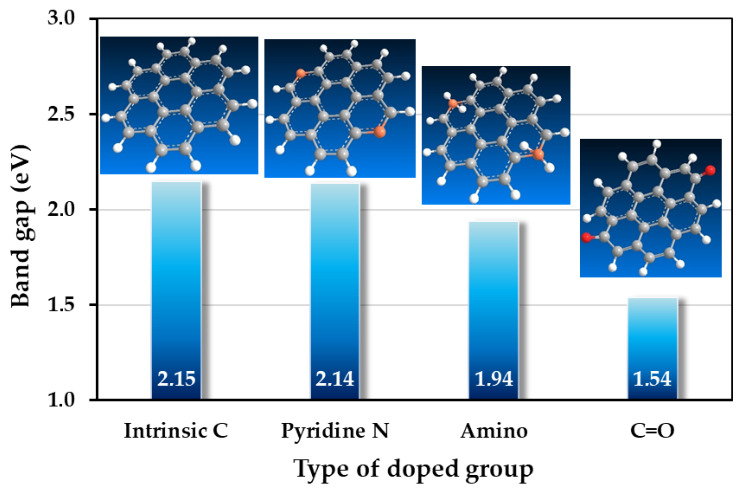
Band gap change in CDs as a function of the doped groups. Adapted from Ref. [20].

Natural and organic carbon sources are used for the synthesis of CDs due to their easy availability, low cost, and environmentally friendly nature. The carbon source also influences the nature of CDs and their potential applications, as shown in Figure 2. Researchers have used fruits like bananas [7], sugarcane [8], apples [9], lemons [10], or grapes [11] and other organic sources like milk, eggs [12], honey [13], onion [14], turmeric [15], and human waste like human hair [16]. Figure 3 shows two different approaches for synthesizing CDs. In the top-down approach, large carbon materials are broken down into CDs, where they used with coal, graphene, etc. The bottom-up approach involves the synthesis of CDs from atoms or molecules [17].

Researchers have synthesized CDs from naturally occurring organic materials, such as fruits, vegetables, and animal waste. Vaibhav Kumar N. Mehta et al. reported that, using a hydrothermal method to synthesize CDs from apple juice, the CDs obtained are (4.5 ± 1.0 nm) in size and emit light in the blue region. They sliced the apples into pieces and made a solution by adding water and then used that solution for CD synthesis [9].

Cindy Dias et al. [18] reported on the synthesis of CDs from various widely cultivated fruits: kiwi, avocado, and pear. They examined the in vitro cytotoxicity and anticancer potential of CDs by comparing them against human epithelial cells from normal adult kidneys and colorectal adenocarcinoma cells. In vivo toxicity tests were conducted on zebrafish embryos due to their unique embryogenesis, where ex utero development with transparent embryos allows for real-time observation. Both in vitro and in vivo studies showed that the synthesized CDs displayed toxicity only at concentrations of ≥1.5 mg mL^−1^. Kiwi CDs demonstrated the greatest toxicity to both cell lines and zebrafish embryos, with lower LD_50_ values. Interestingly, while black pepper CDs caused less cytotoxicity in normal cells than other CDs, they exhibited higher in vivo toxicity. The biodistribution of CDs in zebrafish embryos after uptake was analyzed using fluorescence microscopy, showing increased accumulation of CDs in the eye and yolk sac, with avocado CDs being more retained, suggesting their potential for bio-imaging applications.

They used CDs as fluorescent probes for the imaging of *M. tuberculosis* bacteria, showing confocal microscopic images of *M. tuberculosis* bacteria when they are cultivated with CDs. Without CDs, *M. tuberculosis* does not show any fluorescence while they can show blue, green, and red emissions depending on excitation wavelength. These non-toxic CDs present amino groups and do not show any toxicity, which means that only functional groups are not responsible for the toxicity of CDs.

Kui Chen et al. synthesized CDs from waste tea by hydrothermal method and found that the scavenging capacity of CDs (which influences antioxidant properties of CDs) increases with increasing concentrations of CDs and acts as a biocompatible material; therefore, a hypothesis can be developed from here, that the functional groups are not the reason for the toxicity of CDs [19].

## 3. Key Determinants of Carbon Dot Cytotoxicity

Carbon dot cytotoxicity is not an intrinsic material property, but an emergent outcome governed by multiple interdependent physicochemical and experimental parameters.

### 3.1. Physicochemical Heterogeneity as an Inherent Feature of Carbon Dots

Carbon dots are not a chemically uniform nanomaterial, but rather a heterogeneous ensemble whose physicochemical properties strongly depend on precursor selection and synthesis conditions. Variations in particle size distribution, surface chemistry, degree of oxidation, and structural order are frequently observed even among CDs synthesized using nominally similar protocols. This inherent heterogeneity represents a fundamental challenge for toxicity assessment, as biological responses are often highly sensitive to subtle differences in material properties.

Importantly, many toxicity studies report results for a single CD formulation without sufficient characterization or comparison across batches, implicitly assuming material uniformity. Such assumptions can lead to misleading generalizations and contribute to the wide range of reported cytotoxicity outcomes.

### 3.2. Particle Size, Dimensionality, and Cellular Interactions

Particle size plays a critical role in determining cellular uptake pathways, intracellular localization, and clearance mechanisms. Smaller CDs typically exhibit enhanced cellular internalization, which may increase their interaction with intracellular organelles and elevate the likelihood of oxidative stress or mitochondrial dysfunction. Conversely, larger or aggregated particles may preferentially interact with the cell membrane or become sequestered in endolysosomal compartments.

Beyond size alone, dimensionality represents a key distinction between quasi-zero-dimensional carbon dots and two-dimensional graphene quantum dots. Differences in geometry influence membrane interactions, π–π stacking behavior, and protein adsorption, which in turn affect cellular responses. Failure to explicitly consider these distinctions may obscure material-specific toxicity trends.

### 3.3. Surface Chemistry, Functionalization, and Charge Effects

Surface chemistry is among the most influential determinants of CD cytotoxicity. Functional groups introduced during synthesis (Figure 2) or post-synthetic modification govern colloidal stability, protein corona formation, and interactions with biological membranes. Positively charged or highly reactive surface functionalities are frequently associated with increased membrane disruption and cytotoxicity, whereas neutral or negatively charged surfaces often exhibit improved biocompatibility.

Doping with heteroatoms such as nitrogen, sulfur, or boron can further modulate electronic structure and redox activity, often in a manner that is intrinsically linked to precursor chemistry rather than deliberate post-synthetic modification. While such modifications are commonly employed to enhance photoluminescence or catalytic performance, they may also alter reactive oxygen species generation and biological reactivity, underscoring the need for the careful evaluation of functionalized CDs. In addition to post-synthetic functionalization, the surface chemistry of carbon dots is strongly influenced by the chemical nature of the precursor. In particular, heteroatom doping—most commonly nitrogen—often originates directly from nitrogen-containing precursors and is incorporated during the bottom-up synthesis process. Such precursor-derived doping can simultaneously modify electronic structure, surface charge distribution, and redox activity, thereby exerting a profound influence on biological interactions.

Figure 2 schematically illustrates how precursor composition governs nitrogen incorporation, surface functional groups, and resulting biological responses of carbon dots. This conceptual framework highlights that doping effects cannot be treated as isolated variables, but rather emerge from the coupled relationship between precursor chemistry, synthesis conditions, and surface reactivity. The schematic representation highlights precursor-derived functional groups, nitrogen doping in carbon dots, and their implications for surface chemistry and biological responses. Nitrogen-containing precursors introduce heteroatoms during bottom-up synthesis, leading to modifications in surface functional groups, electronic structure, and charge distribution. These changes can influence protein adsorption, cellular interactions, and redox behavior, ultimately affecting observed cytotoxicity and biocompatibility. Figure 2 emphasizes the interconnected nature of precursor selection, doping, and biological outcomes rather than treating nitrogen doping as an independent design parameter.

CD properties change upon doping, which can alter surface functional groups. Jingjing Yu et al. synthesized CDs using a simple microwave synthesis with glycine as a precursor [20]. Amino groups on the surface are predominant across the entire emission spectrum with a peak at 445 nm, while other emissions linked to impurity levels associated with carbon and nitrogen elements, such as C-C/C=C (intrinsic carbon), C-N (graphitic nitrogen), nitrogen-containing heterocycles (pyridine nitrogen), and C=O groups, are found around 305 nm, 355 nm, 410 nm, and 500 nm, respectively. These distinct luminescent properties originate from electron shifts within various trapped states in the band structure, driven by a variety of chemical bonds in the carbon cores or surface functional groups of the carbon dots. The luminescent traits are linked to carbon–nitrogen bonds and surface functional groups on the CDs. Additionally, the excitation and emission spectra show that the optical properties of the CDs can be altered in acidic or basic environments. They supported the claim by theoretical calculations, and a band structure model was suggested, focusing on potential electron transitions from trapped states within the band gap, which explains the distinct luminescent properties of CDs. Furthermore, the synthesized CDs possess a specific ability to detect Fe^3+^ ions selectively among various metal ions due to a fluorescence inner filter effect between Fe^3+^ ions and CDs. Figure 3 shows that the undoped CDs structure has a band gap value of 2.15 eV; while the pyridine nitrogen functional group decreases the band gap to 2.14 eV, the amine (-NH_2_) group further decreases the band gap to 1.94 eV, and the carbonyl (C=O) group decreases the band gap value to 1.54 eV [20].

Marketa Havrdova et al. [21] found that CD cytotoxicity and intracellular tracking are affected by the charge of surface functional groups and the chemical nature of the functional group. They synthesized negative, neutral, and positively charged CDs. Negative charged CDs have carboxylic groups, while the neutral CDs are modified with polyethylene glycol, and positively charged CDs are encapsulated with polyethyleneimine. They used mouse fibroblasts to test the toxicity and found out that negatively charged CDs up to a concentration of 300 mg mL^−1^ did not induce any irregularities in the cell structure, and even caused the high oxidative stress and stimulated proliferation, but did not enter while positively charged. Even CDs with a concentration of 100 mg mL^−1^ can enter the nucleus and disturb the G0/G1 phase of the cell cycle with damage to the other parts of cells. This study illustrates that surface charge not only governs cellular uptake, but also subcellular localization and cell cycle perturbation, underscoring that cytotoxicity cannot be inferred solely from viability assays.

Surface functional groups should not be interpreted as direct toxic determinants. Rather, they modulate emergent physicochemical properties such as surface charge density, colloidal stability, and redox reactivity. These secondary properties influence membrane interaction, intracellular uptake, and oxidative stress generation. Therefore, apparent contradictions in the literature arise when functionalization is discussed as an isolated chemical feature instead of as a regulator of coupled physicochemical parameters.

### 3.4. Dose, Exposure Conditions, and Experimental Context

Reported toxicity outcomes are strongly dependent on dose, exposure duration, and the biological model employed. Many studies rely on short-term, high-dose exposures that may not reflect realistic application scenarios. Moreover, concentration-dependent transitions between apparent antioxidant behavior and pro-oxidant toxicity have been reported, suggesting non-linear dose–response relationships.

Experimental context, including cell type, culture medium composition, and exposure route in vivo, further complicates direct comparison between studies. Without standardized reporting of these parameters, it remains difficult to establish generalizable toxicity thresholds for CDs.

### 3.5. Methodological Limitations and Assay Interference

An often-overlooked factor in CD toxicity assessment is the potential for assay interference. The intrinsic fluorescence and light absorption properties of CDs can interfere with commonly used colorimetric and fluorometric assays, leading to false-positive or false-negative results. Additionally, adsorption of assay reagents or serum proteins onto CD surfaces may alter assay performance and biological responses.

Failure to account for these methodological limitations can significantly distort toxicity data and contribute to conflicting conclusions across the literature.

### 3.6. Synthesis-Dependent Variability and Reproducibility

Minor variations in synthesis parameters such as precursor composition, reaction temperature, heating rate, solvent environment, and purification procedures can result in substantial differences in the physicochemical properties of carbon dots. Even when nominally identical protocols are employed, subtle changes in experimental conditions may lead to variations in particle size distribution, surface functional group density, heteroatom incorporation, and residual molecular species. Such synthesis-dependent variability is an inherent feature of bottom-up carbon dot fabrication and represents a critical yet frequently underreported determinant of biological response.

From a toxicological perspective, this variability has profound implications. Biological systems often respond sensitively to small changes in surface chemistry, charge distribution, and redox activity, meaning that two batches of carbon dots synthesized under slightly different conditions may elicit markedly different cytotoxicity outcomes. As a result, discrepancies reported across toxicity studies may not necessarily reflect contradictory biological behavior but rather the differences in material identity that are insufficiently captured by routine characterization.

Importantly, most toxicity studies implicitly assume batch uniformity and material reproducibility, despite the absence of standardized synthesis and quality control criteria for carbon dots. In many cases, conclusions regarding cytotoxicity or biocompatibility are drawn from single-batch experiments without an assessment of inter-batch variability. This practice limits the reliability and generalizability of safety conclusions and complicates cross-study comparisons.

These observations underscore the necessity of shifting from material-specific toxicity claims toward a framework that explicitly accounts for synthesis history and variability. Without such a shift, the accumulation of isolated toxicity reports risks reinforcing apparent inconsistencies rather than resolving them. Addressing synthesis-dependent variability is therefore essential not only for reproducible research, but also for the development of data-driven approaches such as machine learning approaches, which rely on consistent and well-annotated input data to identify governing trends in carbon dot toxicity.

### 3.7. Limitations of Viability-Based Cytotoxicity Assessment

A substantial proportion of studies summarized in Table 1 and Table 2 rely primarily on metabolic viability assays such as MTT, CCK-8, and WST-1. While these assays are widely used and experimentally convenient, they measure mitochondrial reductase activity rather than direct cell survival. Consequently, they reflect metabolic perturbation rather than definitive cytotoxicity.

This limitation is particularly relevant for carbon dots, which are intrinsically fluorescent and frequently exhibit redox activity. Several interference mechanisms must be considered:

− Optical interference: Intrinsic fluorescence or absorbance of CDs may overlap with assay detection wavelengths.
− Redox interference: CDs can directly reduce tetrazolium salts, leading to false-positive viability signals.
− Adsorption artifacts: Interaction of nanomaterials with assay reagents may alter colorimetric readouts.
− ROS modulation: Transient oxidative stress may alter metabolic activity without inducing irreversible cell death.

**Figure 4:**
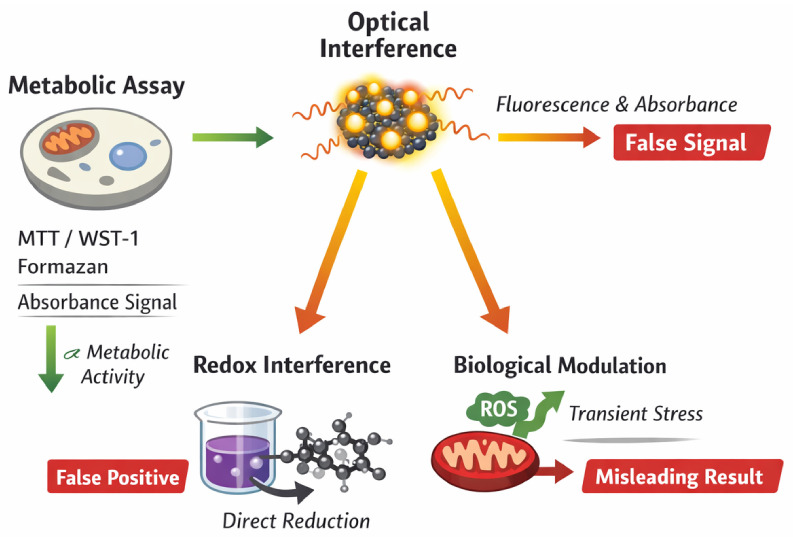
Conceptual mechanisms of interference in metabolic viability assays applied to carbon dot toxicity evaluation.

The limitations of metabolic viability assays in carbon dot research are summarized schematically in Figure 4. Tetrazolium-based assays (MTT, WST-1, CCK-8) are designed to quantify mitochondrial reductase activity, which is commonly interpreted as a proxy for cell viability. However, in CD systems, several interference pathways may distort this relationship.

First, optical interference may occur due to the intrinsic fluorescence or absorbance of carbon dots overlapping with assay detection wavelengths. This can artificially elevate or suppress the measured signal. Second, CDs exhibiting redox activity may directly reduce tetrazolium salts in the absence of viable cells, leading to false-positive viability results. Third, biological modulation of mitochondrial activity—such as transient ROS generation or metabolic stress—may alter enzymatic activity without inducing irreversible cell death. In such cases, reduced metabolic activity does not necessarily correspond to true cytotoxicity.

These mechanisms highlight that viability percentages obtained from metabolic assays should not be interpreted as definitive measures of biocompatibility. Instead, they should be complemented by orthogonal endpoints assessing membrane integrity, apoptosis, oxidative stress, and long-term proliferation.

As a result, viability percentages alone cannot be interpreted as comprehensive indicators of biocompatibility. Complementary endpoints—such as membrane integrity assays (LDH release), apoptosis markers (caspase activation), ROS quantification, mitochondrial membrane potential, and long-term proliferation assays—are required to establish mechanistic understanding.

The predominance of metabolic assays in the current literature contributes to variability in reported toxicity outcomes and may partially explain discrepancies between studies.

Tetrazolium-based assays quantify mitochondrial reductase activity via formazan formation and absorbance measurement. In CD systems, three major interference pathways may distort the readout: (i) optical interference due to intrinsic fluorescence or absorbance of carbon dots, (ii) direct redox interaction between CDs and assay reagents resulting in false-positive signals, and (iii) biological modulation of mitochondrial metabolism through ROS generation or transient stress responses. These mechanisms illustrate why metabolic activity should not be equated directly with cell viability in nanomaterial studies.

### 3.8. Cell-Model–Dependent Toxicity Patterns: Statistical Normalization of the Evidence Base

The data compiled in Table 1 and Table 2 were subjected to atomization prior to statistical analysis. Several publications evaluated multiple cellular models or tested multiple concentration ranges within a single study. To enable cross-study comparison and avoid the double-weighting of publications, each unique combination of carbon dot formulation, cell model, and experimental condition was treated as an independent analytical unit. This atomization approach enabled construction of a harmonized dataset comprising 115 analytical units across 43 distinct cellular models, while preserving the original biological context of each report.

To harmonize the heterogeneous literature reports, viability outcomes were classified into three operational categories: non-toxic (≥80% viability), moderate (50–79% viability), and toxic (<50% viability) effects. When exact numerical values were not reported, classification followed the interpretation given by the original authors; for example, descriptions such as “significant cytotoxicity” were categorized as toxic. This normalization framework was intended to support cross-study comparison in a highly heterogeneous evidence base, and should therefore be interpreted as an exploratory analytical approach rather than a universal toxicity standard.

**Figure 5:**
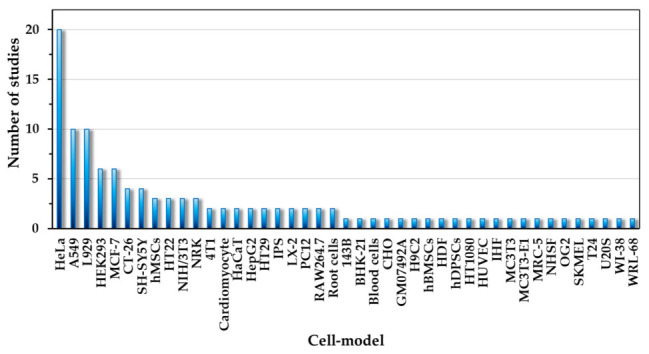
Frequency distribution of cellular models used in carbon dot cytotoxicity studies.

Figure 5 presents the frequency distribution of the cellular models represented in Table 1 and Table 2. A pronounced dominance of the HeLa cell line is observed, followed by A549, L929, MCF-7, and HEK293, whereas many other models appear only once or twice. This uneven distribution has important implications for interpretation of the perceived biocompatibility of carbon dots. When a limited subset of resilient or well-characterized models dominates the literature, broad conclusions regarding “low toxicity” may disproportionately reflect outcomes in those specific systems rather than universal biological safety.

To minimize statistical noise associated with very small sample sizes, models represented by fewer than five independent analytical units were excluded from inferential analysis. This filtering step reduced distortion arising from sparsely represented models, in which a single study could produce an artificial 100% toxicity or 0% toxicity rate. The filtered dataset therefore retained the most frequently studied and statistically meaningful cellular models, enabling the evaluation of systematic trends rather than isolated observations.

Given the heterogeneity of experimental designs, reporting standards, and biological models across the literature, the present analysis is intended to identify comparative patterns rather than to establish definitive quantitative toxicity thresholds.

A wide variety of cellular models has been used in the literature. However, only a limited number of models are represented disproportionately often. Therefore, there is a risk that toxicological test results may be biased by the preferential selection of the HeLa cells, which may demonstrate relatively high tolerance resistance.

Using the filtered dataset (*n* ≥ 5 per model), the relationship between study frequency and reported cytotoxicity was statistically evaluated.

Two toxicity definitions were examined:

Strict toxicity: Only cases classified as Toxic (<50% viability).

Broad toxicity: Moderate + Toxic combined (<80% viability).

**Figure 6:**
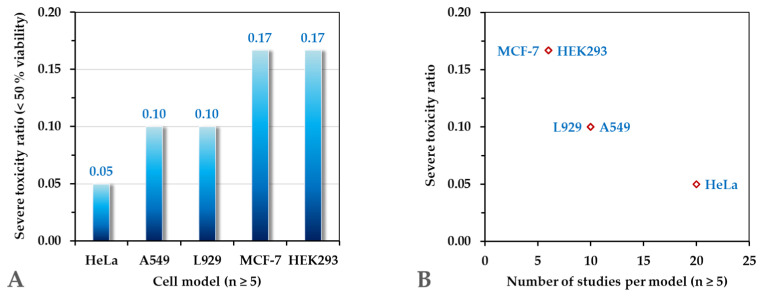
Model-dependent patterns in reported carbon dot cytotoxicity. (**A**) Toxicity ratio (<50% viability) across cellular models represented by ≥5 independent analytical units. (**B**) Relationship between study frequency and severe cytotoxicity ratio (strict definition). A significant negative correlation (r = −0.93, *p* = 0.021) indicates that more frequently studied models report fewer cases of pronounced cytotoxicity.

Under the strict definition, a strong negative correlation was observed between the number of studies per model and the fraction of studies reporting severe cytotoxicity (Pearson r = −0.93, *p* = 0.021). The obtained results are shown in Figure 6A,B.

This finding indicates that more frequently studied cellular models tend to show fewer reports of pronounced cytotoxicity (<50% viability).

In contrast, when moderate effects were included in the toxicity definition, the correlation was weaker and not statistically significant (r = −0.55, *p* = 0.335). This suggests that the observed frequency-dependent effect primarily concerns severe cytotoxic responses rather than sublethal metabolic perturbations.

Importantly, when HeLa was compared directly to all other models using a chi-square test (broad toxicity definition), no statistically significant difference was detected (*p* = 0.596). Therefore, the effect does not appear to be attributable to a single dominant cell line, but rather reflects a system-level pattern within the literature.

The data do not support the simplistic conclusion that CQDs are universally non-toxic due to exclusive reliance on a single resistant model. However, the significant negative correlation observed under the strict toxicity definition suggests a more nuanced interpretation:

Cellular models that are frequently employed in the literature tend to exhibit lower reported rates of severe cytotoxicity.

Several non-mutually exclusive explanations may account for this observation:

- Preferential selection of robust or well-characterized cell lines;
- Optimization of dosing ranges in commonly used models;
- Publication bias against strongly toxic formulations in widely studied systems;
- Adaptive resistance characteristics of immortalized cancer cell lines.

Collectively, these findings indicate that the perceived biocompatibility of carbon dots cannot be interpreted independently of the biological model context. Rather than reflecting intrinsic universal safety, reported toxicity profiles emerge from an interaction between nanomaterial properties and model-specific biological resilience.

This reinforces the need for diversified biological testing and careful normalization of cross-study comparisons.

### 3.9. Toward Minimal Reporting Standards for Carbon Dot Toxicity Studies

Particular attention should be paid to size characterization, as carbon dots typically fall within the 1–10 nm range where quantum confinement effects dominate. At this scale, discrepancies between transmission electron microscopy (TEM) and dynamic light scattering (DLS) are not merely technical artifacts, but reflect fundamentally different physical quantities.

TEM provides a direct visualization of the inorganic core under high-vacuum conditions. However, at sizes around 3–5 nm, an uncertainty of ±1 nm corresponds to approximately 20% of the total size range defining carbon dots. This significantly affects size classification and cross-study comparability. Moreover, image-based particle analysis inherently involves the manual or semi-automatic selection of analyzable particles, which may unintentionally exclude agglomerates or poorly resolved structures.

In contrast, DLS measures hydrodynamic diameter in solution, which includes the solvation shell and any loosely associated surface-bound species. Consequently, DLS values are systematically larger than TEM core sizes. Additionally, DLS is highly sensitive to larger aggregates due to intensity-weighted scattering, often resulting in broader apparent size distributions. Unlike TEM analysis, DLS does not easily allow for the selective exclusion of agglomerates, and small fractions of larger particles may dominate the measured distribution. Therefore, TEM and DLS data should not be interpreted as interchangeable metrics. Reporting both core size and hydrodynamic diameter—together with polydispersity index and measurement conditions—is essential for meaningful interpretation. Without such clarification, apparent discrepancies in reported CD size may reflect methodological differences rather than true material variability.

The variability observed across CD toxicity studies is strongly influenced by inconsistent physicochemical characterization and incomplete reporting. Without standardized descriptors, cross-study comparison remains inherently limited.

Based on the analysis presented in this review, we propose a minimal reporting checklist that would substantially improve reproducibility and interpretability:

Physicochemical characterization:

− TEM size distribution (mean ± SD, number of particles analyzed).
− Hydrodynamic diameter (DLS) and polydispersity index.
− Zeta potential under biologically relevant conditions.
− XPS-based quantification of heteroatom content.
− FTIR or complementary surface chemistry analysis.
− Clear description of purification procedures (e.g., dialysis cutoff, chromatography).
− Verification of removal of small molecular fluorophores.

Biological testing parameters:

− Dose normalization (μg/mL and, when possible, surface area normalization).
− Exposure time.
− Serum conditions.
− At least one orthogonal cytotoxicity endpoint beyond metabolic assays.
− Explicit control experiments for assay interference.

Such minimal standardization would not eliminate biological variability, but would enable meaningful mechanistic comparison and reduce apparent contradictions in the literature.

Since the surface area-to-volume ratio and colloidal behavior are size-dependent, inconsistent size reporting directly propagates into biological interpretation.

## 4. In Vitro Toxicity of Carbon Dots: Endpoints, Pitfalls, and Interpretability

### 4.1. Commonly Used In Vitro Models and Exposure Scenarios

In vitro toxicity of carbon dots has been evaluated using a wide range of cellular models, including immortalized cancer cell lines (e.g., HeLa, A549, HepG2), primary mammalian cells, and, less frequently, stem cell-derived systems. These models are typically selected based on convenience, robustness, and relevance to intended applications such as bioimaging or drug delivery.

However, the choice of cellular model and exposure conditions varies widely across studies, often without explicit justification. Differences in membrane composition, metabolic activity, antioxidant capacity, and endocytic pathways among cell types can significantly influence cellular responses to carbon dots. Consequently, toxicity outcomes reported for one cell line may not be directly transferable to others, limiting the generalizability of isolated findings.

Markéta Havrdová et al. examined the intracellular mobility and distribution of nanomaterials, focusing on their impact on the nuclear region of living cells [22]. They performed comprehensive assessments of cytotoxicity and DNA damage, as well as a detailed analysis of the distribution of CDs in two healthy mouse cell lines, NIH/3T3 and L929, using fluorescent microspectroscopy. Real-time observations were made of CDs crossing the plasma cell membrane, with uptake levels linked to concentration, and endocytic pathways were identified. They discovered that a CDs concentration of 200 µg/mL is near saturation, and in NIH/3T3 cells, this led to a different cell cycle profile without significant changes in viability or DNA damage, even when CDs were present in the nuclei. In contrast, the presence of CDs in the nuclei of L929 cells triggered cell death. The intranuclear environment of NIH/3T3 cells altered the fluorescent characteristics of CDs, causing the fluorescence to shift to blue. Investigating intracellular interactions with CDs is crucial for the advancement of future applications like DNA sensing, as CDs have not yet been developed as DNA probes. Positively charged CDs interacted with mouse fibroblast cells. NIH/3T3 cell response showed that some CDs escape from the endosome and integrate into the cytoplasm while the cell remains alive and CDs do not cause significant damage. In L929 cells, CDs could not escape and accumulate inside, leading to a high intracellular charge concentration and causing cell death, showing that different fibroblast cell lines can react with the same type of CDs differently.

### 4.2. Cytotoxicity Endpoints: What Is Measured and What Is Missed?

Observation of high biocompatibility through standard metabolic assays is consistently reflected across a wide array of the recent literature, as summarized in Table 1. Most in vitro studies assess CD toxicity using a limited set of standard assays, including metabolic activity-based tests (MTT, WST-1, CCK-8), membrane integrity assays (LDH release), and viability staining. While these methods provide useful initial screening information, they primarily capture acute cytotoxic effects and may overlook subtler or delayed biological responses.

Importantly, many studies rely on single-endpoint measurements at a single time point, often at relatively high particle concentrations. Such experimental designs can obscure non-linear dose–response relationships and fail to distinguish between transient cellular stress and irreversible cytotoxic damage. As a result, reported conclusions regarding “low toxicity” or “high biocompatibility” may reflect assay limitations rather than true biological safety.

Lin Li et al. [23] synthesized CDs by employing the hydrothermal method and using red thiourea as a precursor. CDs showed low toxicity with good biocompatibility. They investigated the toxicity of CDs by using HeLa cells as a model in the standard MTT assay to check the concentration of CDs up to 400 μg/mL. The assay showed a cell survival rate up to 80% with low toxicity, suggesting a possible use of CDs in bioimaging.

**Table 1:** Comprehensive summary of Carbon Dot (CD) precursors, synthesis parameters (including methodology, temperature, and duration), and their respective toxicological profiles.

CDs Precursor	Synthesis *	Cells Type	Method	% of Viability	Mechanism of CDs Toxicity	Ref.
Coffee beans	Roasted in the oven at 210 °C for 5, 10 and 20 min	NRK	flow cytometry	Above 85%	CDs induced lysosomal-dependent cell death and increased the rate of cell death through necroptosis	[24]
Spinach (Spinacia oleracea)	Microwave irradiation/700 W for 5 min	hBMSCs	Cell counting kit-8 (CCK-8) assay	No cytotoxicity	Increase in apoptosis	[25]
Aloe vera extract	Microwave-assisted at 80 W for 1 min	MCF-7	MTT assay	50% at a concentration of 52.2 μg/mL	Anti-proliferative and -apoptosis effect	[26]
Glucosamine hydrochloride, ethylenediamine, ascorbic acid	Hydrothermal 200 °C/4 h	HeLa H9C2	MTT assay MTT assay	65.6% at concentration 100 μL mL^−1^ 71% at concentration 100 μL mL^−1^	Generated ROS inside the cell by unbalancing antioxidant enzyme activity	[27]
Coal and curcumin	Sonicated 3 h followed by heating at 120 °C for 24 h continuous stirring	NIH/3 T3	MTT assay	50% at concentration 750 μg mL^−1^ of cur-GQDs	Generated ROS inside the cell	[28]
baked lamb meat	Carbonization (280 °C (15–45 min)	PC12	Annexin V-FITC/PI double staining flow cytometry	No cytotoxicity >90% viability at 0.5 mg/mL after 24 h of high cytotoxicity (longer baking time)	Higher due to carbonization degree and surface groups	[29]
1-butyl-3-methylimidazolium dicyanamide (BmimDCN)	hydro-thermal method (200 °C—12/48 h) Ethanol-thermal method (200 °C for 12 h)	LX-2 SK-Hep-1	MTT assay ROS assay	Hydrophilic CDs: no cytotoxicity up to 200 μg/mL (>80% cell viability) Hydrophobic CDs: Higher cytotoxicity	Hydrophobic interactions, potentially causing membrane damage	[30]
Citric acid	Solvothermal method (250 °C)	hMSCs	MTT assay WST-1 assay Live/Dead Assay	Low toxicity up to 200 µg/mL (90–97% cell viability) Threshold toxicity at 1 mg/mL with complete cell death at 10 mg/mL	Partial agglomeration in cell culture medium	[31]
Freshly ripened (*Aegle marmelos*) fruit	Hydrothermal method (250 °C for 8 h)	*Allium sativum* (garlic) root meristem cells	optical light microscopy	Non-toxic at 50 ppm Genotoxicity at 100–250 ppm	Chromosomal aberrations Reduced mitotic index	[32]
Chicken blood, ethylene diamine	Hydrothermal carbonization (180 °C for 6 h)	A549 NHSF	Annexin V-FITC (flow cytometry) SRB assay Fluorescence microscopy Caspase-3 ELISA	Significant cytotoxicity against A549 No toxicity towards NHSF (IC50 > 1000 µg/mL)	Apoptosis induced through nuclear damage and caspase-3 activation	[33]
Citric acid, Metformin hydrochloride	Hydrothermal synthesis (200 °C for 12 h).	MCF-7 MDA-MB-231	MTT assay ROS assay	Significant cytotoxicity	Increased ROS and apoptosis through Caspase-3 expression	[34]
Citric acid, Urea	Microwave-assisted synthesis (180 °C for 10 min)	OG2 MC3T3-E1	CCK-8 assay	Cytotoxicity at higher concentrations: >80 µg/mL >40 µg/mL	Apoptosis	[35]

* Synthesis conditions, temperature, and time.

The consistency of high cell viability reported across early studies is further challenged when examining a broader range of biological models and complex precursors, as detailed in Figure 2. A prominent trend emerging from this data is the significant disparity in toxicological outcomes when the same Carbon Dots (CDs) are tested against different cell lines. For instance, CDs derived from palm kernel shells and oyster shells demonstrated high viability in robust HeLa cells (>90% and >60%, respectively), but showed a marked decrease in survival when applied to more sensitive induced pluripotent stem (iPS) cells or cardiomyocytes, where viability dropped to as low as 50–60%. This suggests that the “low toxicity” often reported in standard screenings may be a localized observation rather than a universal trait. Furthermore, Table 2 highlights how the chemical nature of the precursor and the resulting surface charge can dictate biological safety regardless of the synthesis method. While naturally derived sources like bread or orange juice typically yield benign particles even at higher concentrations (up to 1.5 mg/mL), synthetic or nitrogen-rich precursors can introduce significant risks.

**Table 2:** Expanded summary of CD precursors, synthesis conditions, and cell-specific viability outcomes.

CDs Precursor	Synthesis *	Cells Type	Method of Analysis	% of Viability	Ref.
Human tumor lines (most commonly used)					
Palm kernel shell	Carbonized in nitrogen gas for 3 h at 600 °C followed by hydrothermal treatment at 160 °C for 4 h	HeLa, IPS, cardiomyocyte	Cell Counting Kit-8	More than 90% More than 60% More than 70%	[36]
Oyster shell	Carbonized in nitrogen gas for 3 h at 600 °C followed by hydrothermal treatment at 160 °C for 5 h	HeLa, IPS, cardiomyocyte	Cell Counting Kit-8	More than 60% More than 90% More than 50%	[36]
EDTA disodium and sodium sulfide	Hydrothermal/200 °C for 8 h	RAW 264.7 or HeLa	MTT assay	No cytotoxicity	[37]
Citric acid with boric acid, nitric acid, sulfuric acid and phosphoric acid	Furnace-assisted/300 °C and microwave method at 600 W both for 2 h	HeLa	CCK-8 assay	Above 80% up to 500 μg mL^−1^	[38]
Citric acid and p-phenylenediamine and	Hydrothermal 200 °C for 8 h.	HeLa	MTT assay	No cytotoxicity, above 80% up to 500 μg mL^−1^	[39]
Ethylenediamine	Hydrothermal 180 °C for 20 h	HeLa and A549	WST-1 assay	Above 80% up to 1 mg mL^−1^	[40]
Soy sauce and activated silica gel	Silica gel column chromatography/Room temperature	HeLa	MTT assay	No cytotoxicity	[41]
Ammonium citrate tribasic, vitamin B1	Microwave-assisted hydrothermal approach (from 110 to 180 °C at 1200 W for 2 h)	A549 HeLa	MTT assay	Low toxicity (cell viability > 54.1%) at concentration 1000 μg mL^−1^	[42]
Ibuprofen, triethanolamine	One-step microwave-assisted method (700 W for 8 min)	HeLa, BABL/c mice tissues (heart, liver, kidneys)	MTT assay hematoxylin and eosin staining	No cytotoxicity after 24–72 h No toxic effects on tissues	[43]
Tomato and lemon juices	Hydrothermal treatment (160 °C for 3 h)	HeLa	MTT assay	No cytotoxicity (cell viability 73–85%)	[44]
citric acid, thiosemicarbazide	One-pot hydrothermal technique (180 °C for 5 h)	HeLa	MTT assay	No cytotoxicity	[45]
Wet pomace	hydrothermal carbonization (200–300 °C)	L929 HeLa	PrestoBlue™ assay	No cytotoxicity at 24 h, up to a concentration of 500 μg/mL (>80% cell viability)	[46]
citric acid, ethanolamin	Thermal approach (180 °C for 30 min)	HeLa	MTT assay	Low cytotoxicity with high cell viability (86–95%) at concentrations up to 200 μg/mL	[47]
Citric acid, *p*-phenylenediamine	One-pot hydrothermal synthesis (200 °C for 8 h)	HeLa	MTT assay	No citotoxicity (>80% cell viability) at concentrations up to 500 µg/mL	[39]
*L-Tartaric acid*, *o-Phenylenediamine*	Solvothermal synthesis (200 °C for 6 h)	HeLa	MTT assay fluorescence microscopy	No cytotoxicity (~89% viability) at 2.5–6.5 mg/mL Significant cytotoxicity at >12.5 mg/mL	[48]
Diaminomaleonitrile, Boc-D-2,3-diaminopropionic acid	Hydrothermal synthesis (200 °C for 5 h)	HeLa	MTT assay	Cell viability > 90% at 200 µg/mL	[49]
Tulsi (*Ocimum tenuiflorum*) leaves	Carbonization method (300 °C for 3 h)	HeLa	XTT assay	No citotoxicity Cell viability: 95% at 0.05 μM; 78% at 2 μM	[50]
Congo red, Citric acid	Muffle furnace heating (180 °C for 5 h)	HeLa HEK293	MTT assay, Thioflavin T assay	>80% viability at ≤100 µg/mL ~70–90% viability at 100 µg/mL	[51]
Para-phenylenediamine	Hydrothermal synthesis (180 °C for 4 h)	HeLa	MTT assay	No cytotoxicity, cell viability > 88% at concentrations ≤ 300 µg/mL	[52]
Glucose monohydrate and ethylenediamine	Hydrothermal, 180 °C/3 h	A549	Real-Time Cell Analysis of Impedance technique	No cytotoxicity at concentration 1.5 mg/mL and lower	[53]
Citric acid and gold nanoparticles	Hydrothermal/ 160 °C for 2 h	A549 or HDF	MTS metabolic catalytic assay	No cytotoxic activity on healthy or cancer cell lines	[54]
Citric acid and ethylenediamine	Hydrothermal, 200 °C/1 h	A549	MTT assay	No cytotoxicity	[55]
Melia azedarach leaves	Hydrothermal 220 °C/18 h	A549	MTT assay	No cytotoxicity	[56]
Gelatin	Hydrothermal	A549	Viability (24 h)	~240 µg/mL, ~90% viability	[57]
Chitosan/gamma-Al_2_O_3_/CDs nanocarrier	Hydrothermal 180 °C/12 h	MCF-7	MTT assay	52%	[58]
Citric acid, magnesium hydroxide, ethylenediamine	Hydrothermal/ 200 °C for 4 h.	MCF-7 or 4T1 or CHO	Neutral red	Upper than 60% low toxicity for all three cell lines at a concentration of 2 mg/mL after 24 h	[59]
Fresh leaves of Mexican Mint	Microwave irradiation for 4–7 min at 1200 W	MCF-7	MTT assay	Low cytotoxicity	[60]
citric acid, urea	Solvothermal treatment	GM07492A, MCF-7	MTT assay	No cytotoxicity after 48 h (at high doses of 800 μg mL^−1^ cell viability > 90%)	[61]
raw detonation nanodiamonds (NDs)	Laser ablation in liquid (LAL)	CNE2 HepG2 NP69 RAW264.7 BALB/c peripheral blood BALB/c liver	CCK-8 assay Annexin V/PI staining flow cytometry flow cytometry (ROS levels) RNA analysis	No cytotoxicity at 12, 24, 48 h No toxicity at the concentration of 10 mM no immunotoxicity minimal immunotoxicity	[62]
Red pomegranate peels	Hydrothermal 170 °C/12 h	HepG2 NIH/3 T3	MTT assay	No cytotoxicity	[63]
1,2-Naphthoquinone sulphonate, Ethylenediamine	Self-exothermic reaction (room temperature for 3)	HepG2	MTT assay	No citotoxicity, cell viability > 88% at 0.025 mg/mL	[64]
Iohexol	Hydrothermal 180 °C for 8 h	HT29	MTT assay	No cytotoxicity, above 80% up to 300 μg mL^−1^	[65]
Graphite pellet	Pulsed laser ablation (PLA)-25 °C	SH-SY5Y	CCK-8 assay	Low cytotoxicity at lower concentrations (increasing cell viability from 34% to 55.3%.)	[66]
Curcumin powder	Pyrolysis and alkaline treatment (120, 150, 180, or 210 °C for 2 h).	SH-SY5Y	CCK-8 assay	No cytotoxicity (>90% cell viability) up to 200 µg/mL	[67]
Red cabbage	One-step hydrothermal (220 °C for 36 h)	SH-SY5Y	CCK-8 assay	No cytotoxicity (at 0.5 mg/mL cell viability > 80%)	[68]
Gelatin	Hydrothermal method (200 °C for 3 h).	SH-SY5Y	Live/Dead assay	No cytotoxicity high cell viability up to 5 mg/mL	[69]
Natural osmanthus fragrans	Hydrothermal/180 °C for 10 h	T24	MTT assay	No cytotoxicity	[70]
Hydroxyethylidene diphosphonic acid, urea, and formamide	Hydrothermal 180 °C/12 h	U2OS or 143B	MTT assay	No cytotoxicity	[71]
Terminalia chebula powder, Ethylenediamine.	Hydrothermal method (180 °C for 6 h)	SKMEL L6	MTT assay	Significant toxicity with dose-dependent viability reduction >94.5% viability even at 100 µg/mL	[72]
Mouse tumor lines					
Three types of bread	Toasting for 240 °C/5 min followed by centrifuge	CT-26 or HT29	MTT assay	No cytotoxicity (more than 95% of cells survived even at the highest concentration tested (1.5 mg/mL))	[73]
Riboflavin and ethylenediamine	Hydrothermal/6 h at 180 °C	4T1	CCK-8 assay	No cytotoxicity	[74]
Non-cancer lines/fibroblasts/epithelial cells					
Citric acid and ethylenediamine	Hydrothermal, 180 °C/6 h	L929	MTT assay	No cytotoxicity up to 100 μg/mL	[75]
Tris(hydroxymethyl)aminomethane (Tris)—betaine hydrochloride salt	Thermal oxidation/250 °C	NIH/3T3 or L929	LIVE/DEAD^®^ Viability/Cytotoxicity Kit	No cytotoxicity	[22]
Citric acid	Pyrolysis/200 °C for 1 h	L-929	MTT assay	No cytotoxicity More than 80% at concentration of 100 μg	[76]
Citric acid, 1,5-diaminonaphthalene	One-pot solvothermal method	L929	MTT assay	No cytotoxicity after 24 h (cell viability > 95%)	[77]
Glucose, glucosamin, cellulose and chitosan	Stainless-steel hydrothermal reactor (180 °C for 12 h)	L929	XTT assay	No cytotoxicity (cell viability > 70%)	[78]
Human hair biowaste	Microwave synthesis (180 °C 5–7 min).	L929	MTT assay	No citotoxicity (>99.47% cell viability)	[79]
Glucose	Hydrothermal method (180 °C for 12 h)	L929	MTT assay	No cytotoxicity, upper than 90% cell viability at a concentration up to 0.5 mg/mL after 72 h	[80]
Leaf extract of *Hibiscus rosa-sinensis Linn*	Microwave-assisted synthesis (121 °C for 20 min).	L929 HaCaT	Annexin V/PI apoptosis assay flow cytometry	~28% viability at 500 μg/mL ~60% viability at 1 mg/mL	[81]
Garlic, red lentils	Microwave-assisted synthesis (3 min)	WI-38	MTT assay	No citotoxicity, viability ~78.6% at 25 μg/mL.	[82]
Citric acid, biogenic amine	Microwave reaction system (130 °C for 20 min at 300 W)	HaCaT HEK-293	MTT assay	No cytotoxicity after 24 h at 500 μg/mL	[83]
Fresh mackerel meat	Extraction (230 °C for 30 min)	NRK	MTT assay	No citotoxicity (>90% cell viability) at concentrations <10 μg/mL 44.8% cell viability at 10 mg/mL	[84]
Curcumin	Pyrolysis (180 °C for 2 h)	BHK-21	MTT assay	No cytotoxicity, over 80% cell viability at 100 µg/mL concentration (50 times lower than native curcumin)	[85]
Organ-derived/specialized cells					
Spermidine trihydrochloride	Ultrasound procedure in ice at 500 W 60 min at 4 °C	HUVEC	Live/Dead Cell Double Staining Kit	No significant cytotoxicity	[86]
Cow milk	Time from 1 to 8 h, hydrothermal method, temperature 190–200 °C	HT22	N/A	Survival level of 20% for 40 mg/mL dosage and up to 80% for dosage of 0.4 mg/mL	[87]
Orange pericarp	Hydrothermal/4 h at 140 °C	CT-26	MTT assay	Concentrations from 0.005273 to 0.084375 mg/mL: viability over 90% Concentration of 0.675 mg/mL: viability over 50%	[88]
Orange juice and AgNO_3_ and NaBH_4_	Hydrothermal/120 °C for 2.5 h	HEK 293	MTT assay	No cytotoxicity (6 μg/mL)	[89]
Black carbon	Microwave-assisted method/600 W for 2 min	CT-26	MTT assay	No cytotoxicity	[90]
Glycyrrhizae Radix et Rhizoma	Calcination of precursor two steps 75 °C within 5 min and maintained for 25 min and in the second step to 375 °C within 25 min and maintained for 1 h	RAW 264.7	CCK-8 assay	No cytotoxicity	[91]
Metformin	Microwave assisted method, 1000 W. for 3 min	HEK-293	MTS assay	No cytotoxicity	[92]
Garlic peel (nutshell)	Hydrothermal 180 °C/4 h	IHF	LIVE/DEAD^®^ Viability/Cytotoxicity Kit MTT assay	No cytotoxicity even at concentration 1000 μgmL^−1^	[93]
Manganese citrate and urea	Hydrothermal 180 °C/10 h	HEK-293	MTT assay	No substantial toxicity	[94]
Spirulina powder	Microwave-assisted method/800 W for 60 min	fibroblast cell line (1929)	MTT assay	No cytotoxicity	[95]
Phoenix dactylifera (Date palm fruit)	Microwave-assisted pyrolysis method (100 °C with an output power of 700 Watts for 6 min)	WRL-68 HT1080 zebrafish embryo	MTT assay Trypan blue dye exclusion assay Kaplan- Meier survival curve Morphological analysis	No cytotoxicity at 24 h and 48 h (cell viability up to 80% for 1000 µg/mL) slight toxicity after 48 h No mortality no changes in morphology	[96]
Fat-free cow milk	Hydrothermal method (190–200 °C)	HT22	PrestoBlue™ assay	Strong cytotoxicity at 40 mg/mL after 1 and 2 h (cell survival less than 20%) no cytotoxicity at 4–20 mg/mL (60–80% cell survival)	[87]
Ground hibiscus flower biomass	Hydrothermal synthesis	peripheral blood cells (Wistar mice)	Comet assay trypan blue staining	Low cytotoxicity Genotoxicity at higher concentrations	[97]
*Setaria viridis* (green foxtail grass)	Hydrothermal synthesis (180 °C for 3 h)	MC3T3-E1 hBMSCs In vivo: C57BL/6 and NOD mice	Live/Dead assay Annexin V/PI apoptosis assay flow cytometry	>95% viability at 1–10 µg/mL no toxicity in mice	[98]
Citric acid	Thermal decomposition (150 °C for 120 min)	hDPSCs	MTT assay	No cytotoxicity Cell viability > 90% at 1 µg/mL concentration	[99]
Diammonium citrate, Spermidine trihydrochloride	Carbonization (180 °C for 2–4 h)	HEK293	CCK-8 assay ROS quantification	Positively charged: significant cytotoxicity at 100 μg/mL Negatively charged: no citotoxicity	[100]

* Synthesis conditions, temperature, and time.

Table 1 and Table 2 are presented as a structured evidence base rather than a meta-analytic dataset. Due to heterogeneity in reporting standards across the literature, parameters such as size distribution, zeta potential, purification procedures, and dose normalization are not consistently available and therefore cannot be uniformly harmonized without introducing subjective reconstruction. Instead, the compiled data were atomized and statistically normalized in Section 3.8.

### 4.3. Reactive Oxygen Species as a Frequent but Context-Dependent Mechanism

Generation of reactive oxygen species (ROS) is among the most frequently reported mechanisms underlying CD-induced cytotoxicity in vitro. Numerous studies associate increased intracellular ROS levels with mitochondrial dysfunction, lipid peroxidation, and apoptosis following CD exposure. At the same time, other reports describe antioxidant or ROS-scavenging behavior of CDs under similar experimental conditions. These apparently contradictory observations highlight the context-dependent nature of ROS-related responses and foreshadow the need for a more nuanced interpretation, which is addressed in detail in a later section of this review. In the context of in vitro assays, differences in CD surface chemistry, doping, concentration, and illumination conditions can all influence redox behavior and ROS measurements.

Li-Na Wu et al. investigated CD biocompatibility by synthesizing a composite of CDs with Levofloxacin, a chemotherapeutic bactericidal drug with strong antimicrobial properties [101]. They used the hydrothermal method to produce a nano-composite and found that the synergic effect from CDs increased the antibacterial properties of Levofloxacin. Further investigations showed that CDs composite can be able to break the membrane of Gram-negative and Gram-positive bacteria. Moreover, reactive oxygen species (ROS) assays showed that CDs can enter the bacteria and produce ROS which changes the normal state of bacteria and killed them.

Meng Zhang et al. developed a new ZnO@CDs conjugated with protein concanavalin A nanoprobe for highly sensitive and selective electrochemiluminescence (ECL) evaluation of leukemia K562 cells [66]. The obtained sensor exhibited good stability and selectivity against a random DNA aptamer that was negative for K562 cells, as a control. One additional interesting observation was that the obtained ZnO@CDs can be used to stain the K562 cells. The results were compared to the calcein AM (green). All the tests were carried out ex vivo; however, cytotoxicity tests have shown that increased concentrations of ZnO@CDs significantly reduced cells viability, shedding light on some of the dangers of CDs.

### 4.4. Assay Interference and Methodological Artifacts

A critical but frequently underestimated issue in the in vitro toxicity assessment of carbon dots is assay interference. The intrinsic fluorescence and light absorption properties of CDs can directly interfere with colorimetric and fluorometric assays, leading to artificially elevated or suppressed signal readouts. Additionally, adsorption of assay reagents or serum proteins onto CD surfaces may alter assay performance and cellular responses.

Failure to account for such artifacts can result in false-positive or false-negative toxicity conclusions. Despite growing awareness of these limitations, many studies do not include appropriate controls to evaluate potential assay interference, further contributing to inconsistencies across the literature.

### 4.5. Interpreting In Vitro Toxicity Data in a Broader Safety Context

While in vitro assays remain indispensable tools for initial toxicity screening, their predictive value for in vivo and human-relevant outcomes is inherently limited. Acute cytotoxicity observed under simplified in vitro conditions does not necessarily translate into adverse effects in complex biological systems, nor does the absence of cytotoxicity guarantee long-term safety.

**Figure 7:**
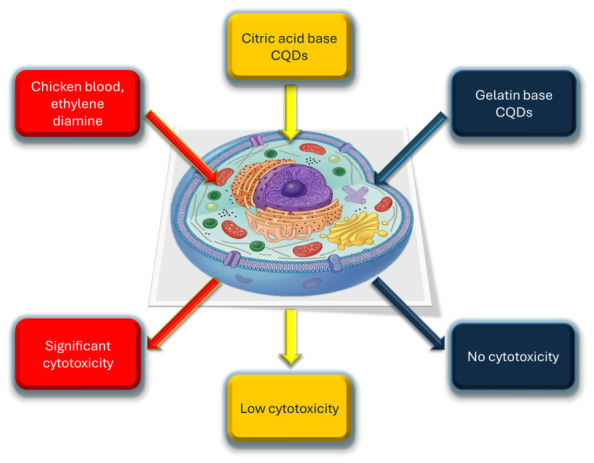
The influence of precursor type on the cytotoxicity of cancer cells.

Given the substantial variability in reported in vitro cytotoxicity outcomes, Figure 7 summarizes the literature data linking precursor type with cellular responses, highlighting both apparent trends and their inherent limitations. In the context of carbon dots, in vitro toxicity data should therefore be interpreted as context-specific indicators rather than definitive safety assessments. Integrating such data with physicochemical characterization, in vivo studies, and mechanistic insights is essential for constructing a coherent and reliable biosafety profile.

## 5. In Vivo Toxicity and Biodistribution of Carbon Dots

### 5.1. Rationale for In Vivo Assessment

In vivo studies are essential for evaluating the biosafety of carbon dots beyond the simplified conditions of in vitro assays. While cellular models provide valuable mechanistic insights, they cannot capture key processes such as biodistribution, metabolism, clearance, immune interactions, or organ-specific accumulation. As a result, in vivo investigations play a critical role in assessing systemic toxicity and translational relevance, particularly for biomedical applications. Nevertheless, the direct comparison and generalization of reported outcomes are impeded by the substantial methodological diversity that characterizes in vivo toxicity data for carbon dots, as is the case with in vitro studies.

Singh et al. (2018) synthesized biocompatible and fluorescent carbon quantum dots (CDs) from beetroot extract to explore their application in in vivo imaging [102]. The authors employed two distinct emission types of CDs—blue- and green-emitting dots—for live animal imaging. The toxicity and distribution of these CDs were assessed in vivo using the nematode *C. elegans* and BALB/c mice. In *C. elegans*, the CDs were introduced via environmental exposure, and their toxicity was evaluated by examining behavior, development, and locomotion, revealing no adverse effects at the tested concentrations. For BALB/c mice, green-emitting CDs (G-CDs) were administered via tail vein injection. The subsequent in vivo biodistribution revealed optical signals predominantly in the lower abdominal region, specifically the intestine, with excretion through feces. Both animal models demonstrated that the CDs were non-toxic and displayed stable and bright fluorescence, supporting their potential use as diagnostic tools in biomedical applications.

Qu et al. (2020) developed bifunctional ibuprofen-based carbon quantum dots (ICDs) using a microwave-assisted method, designed for dual purposes: bioimaging and delivering anti-inflammatory effects [43]. These ICDs demonstrated high stability, negligible toxicity, and excellent biocompatibility. The in vivo evaluation involved administering ICDs intraperitoneally at a dosage of 25 mg/kg to BALB/c mice, comparing them with groups receiving only ibuprofen or PBS (phosphate-buffered saline control). Assessments at 1, 3, and 5 days post-injection included comprehensive hematological and biochemical analyses, alongside histological evaluations of major organs. The results indicate that ICDs did not significantly alter hematological markers such as RBC, WBC, PLT, HGB, or mean corpuscular hemoglobin concentrations. Similarly, biochemical markers related to liver, gallbladder, and kidney function (ALT, AST, ALP, TBIL, BUN, CREA) showed no significant changes compared to control groups, suggesting no toxic impact. Histological examinations of the heart, liver, and kidneys also showed no abnormalities, leading to the conclusion that ICDs are safe for in vivo use and hold potential for clinical applications in bio-imaging, and anti-inflammatory treatments.

In a novel approach, Xu et al. (2016) synthesized fluorescent aspirin-based carbon dots (FACDs) that offer dual functionality for bioimaging and anti-inflammatory treatment [103]. This study utilized a microwave-assisted method to combine aspirin with hydrazine, creating FACDs capable of efficiently penetrating human cervical carcinoma and mouse monocyte macrophage cells with minimal cytotoxicity. Both in vitro and in vivo analyses demonstrated that FACDs provide effective anti-inflammatory effects and exhibit superior biocompatibility compared to aspirin alone. The in vivo biocompatibility was extensively tested on rats, where hematological (RBC, PLT, WBC, HGB, HCT, MCH) and serum biochemistry assays (ALT, AST, ALP, TBIL, BUN, CREA) across multiple days showed no significant adverse effects, confirming that FACDs do not adversely affect blood parameters or liver, gallbladder, and kidney function. Histological analysis also revealed no abnormalities in major organs such as the heart, liver, spleen, and kidneys. These promising results suggest that FACDs could be effectively used in clinical settings for simultaneous imaging and treatment applications without inducing harmful side effects.

In a detailed investigation, Zhao et al. (2021) explored the properties and biological impacts of carbon dots derived from cigarette mainstream smoke (CS-CDs) [104]. The study aimed to assess whether CS-CDs contribute to the anxiolytic effects associated with smoking, using a variety of characterization techniques and behavioral tests in mouse models. The CS-CDs demonstrated a quantum yield of 13.74% and were mainly composed of carbon, oxygen, and nitrogen, with various functional groups identified on their surfaces. The anti-anxiety and sedative effects of these nanoparticles were substantiated using behavioral assessments, such as the elevated plus maze and open-field tests. Additionally, these effects were correlated with neurochemical alterations in the mice, specifically a decrease in glutamate and an increase in norepinephrine in the brain, along with a reduction in serum dopamine levels. Acute toxicity experiments indicated that intraperitoneal injections of CS-CDs at concentrations up to 1420 µg/mL resulted in no significant changes in weight, diet, behavior, or mortality among the treated mice. Furthermore, no pathological changes were observed in major organs post-dissection. These findings suggest that CS-CDs have low toxicity and may modulate neuroendocrine functions, offering a novel perspective on the components of cigarette smoke and potential targets for developing smoking cessation therapies.

Shivam K. et al. (2023) developed a graphene quantum dot (GQD)–polyacrylic acid (PAA) hybrid hydrogel designed as a biomimetic scaffold for accelerated diabetic wound healing [105]. This hybrid hydrogel was evaluated for its anti-inflammatory effects and its ability to promote rapid wound closure, with wounds healing in just 13 days. The research team employed a comprehensive in vivo toxicological analysis to assess the safety of this novel material. Histological assessments of vital organs (heart, liver, kidney, and brain) and wound areas in treated animals showed no significant structural changes or toxicity at any concentration used, indicating excellent biocompatibility. Additionally, hemocompatibility assays, including RBC hemolysis and agglutination tests, further supported the non-toxic nature of the GQD-PAA formulations. These findings underscore the potential of GQD-PAA hybrid hydrogels as safe and effective therapeutic options for managing diabetic wounds, demonstrating their role as a significant advancement in the field of biomaterials and diabetic wound care.

### 5.2. Animal Models Employed in Carbon Dot Toxicity Studies

A wide range of animal models have been used to investigate the in vivo toxicity of carbon dots, including zebrafish embryos and adults, rodents (mice and rats), and, less frequently, invertebrate organisms such as planarians or Caenorhabditis elegans. Each model offers distinct advantages and limitations with respect to throughput, ethical considerations, and biological relevance.

Zebrafish embryos are frequently employed due to their optical transparency, rapid development, and suitability for high-throughput screening. They are particularly useful for evaluating developmental toxicity, cardiotoxicity, and gross morphological abnormalities. However, the extrapolation of zebrafish embryo data to mammalian systems remains limited, as exposure routes, metabolism, and immune responses differ substantially.

Rodent models provide greater physiological relevance for human risk assessment, enabling the evaluation of organ-specific toxicity, biodistribution, hematological parameters, and histopathological changes. Nevertheless, rodent studies are typically conducted using a limited number of doses and time points, often without long-term follow-up, which restricts conclusions regarding chronic exposure or accumulation.

M. Wojnicki et al. have developed gadolinium-doped carbon quantum dots (GCQDs) that act as dual-contrast agents for both fluorescent and magnetic resonance imaging (MRI). These GCQDs are synthesized using hydrothermal treatment, integrating gadolinium into the carbon quantum dot matrix. The resulting nanodots exhibit enhanced magnetic properties compared to gadobutrol, a standard MRI contrast agent [106]. To evaluate the safety of these GCQDs, the researchers conducted toxicity tests using zebrafish embryos (Danio rerio). The studies assessed various concentrations of GCQDs, monitoring mortality, hatchability, malformations, heartbeats, spontaneous movement, and uptake of GCQDs. The results indicate that while survival rates were comparable to control groups, higher gadolinium concentrations in GCQDs led to a significant decrease in hatchability. Fluorescence microscopy showed no statistical differences in fluorescence intensity between groups. These findings suggest that GCQDs could serve as effective dual-contrast agents, combining the optical imaging capabilities of carbon quantum dots with the enhanced MRI contrast provided by gadolinium. However, the study emphasizes the necessity for further research into the synthesis methods and biological interactions of GCQDs to ensure their safety and efficacy in medical applications.

### 5.3. Biodistribution and Clearance Pathways

Biodistribution of carbon dots following in vivo administration is strongly influenced by particle size, surface chemistry, charge, and aggregation state. Many studies report preferential accumulation in organs associated with the reticuloendothelial system, including the liver and spleen, particularly for larger or surface-modified CDs. In contrast, smaller and more hydrophilic CDs are often reported to undergo rapid renal clearance.

Despite these general trends, reported biodistribution patterns vary widely across studies, reflecting differences in material properties, administration routes, and detection methods. In some cases, conclusions regarding clearance or accumulation are based solely on fluorescence imaging, which may be confounded by signal attenuation, tissue autofluorescence, or degradation-related changes in optical properties.

**Figure 8:**
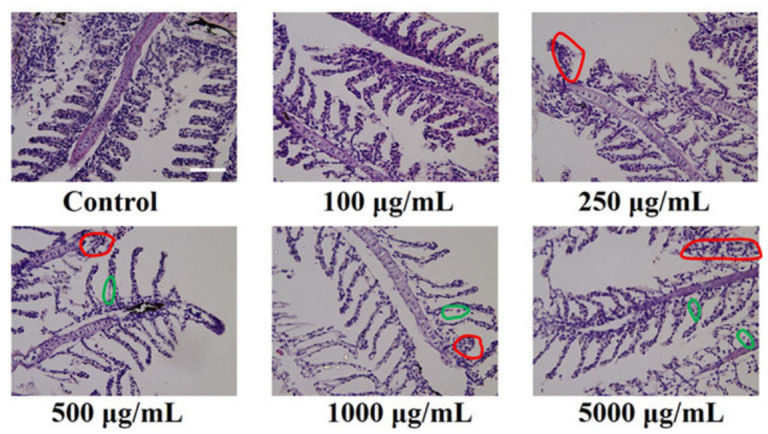
Representative gill histological images. Gill morphology in an adult zebrafish after 14 days of CD exposure. Red circles correspond to secondary lamellae lamellar fusion, while green circles indicate dilated marginal channels. Scale bar: 50 μm. Reprinted from [107].

Wei Liu et al. used sugarcane molasses as a precursor to produce CDs by a hydrothermal method [107]. Due to the uncertain hazards associated with new materials, zebrafish are commonly used to assess toxicity. The results are shown in Figure 8.

A zebrafish model was chosen to test the toxicity of CDs due to its low cost, fast growth, and high genetic homology to humans. Researchers synthesized spherical CDs with a size of 2.4 nm that emitted blue light when excited at 365 nm. The CDs did not show any embryotoxic behavior when used at a concentration of 150 μg/mL, but showed significant toxicity at concentrations above 200 μg/mL. High CDs concentrations affected zebrafish mobility, reduced dopamine levels, and caused damage to multiple organs, which suggests that CD concentration is a significant factor in toxicity. Researchers prepared CD dispersions at concentrations of 50, 100, 150, 200, 250, 300, and 400 μg/mL and exposed zebrafish embryos to them, using 20 embryos per concentration. Figure 8 and other results not presented here confirm that CDs are toxic above a certain concentration.

CD exposure caused morphological changes in zebrafish liver at concentrations above 200 μg/mL. In Figure 8, red circles show pyknotic nuclei (programmed cell death), green circles indicate swollen cells at concentrations above 500 μg/mL, and yellow circles indicate hepatocyte necrosis (dead cell).

### 5.4. Reported In Vivo Toxicity Outcomes

Most in vivo studies report on the low acute toxicity of carbon dots at experimentally tested doses, with minimal changes in body weight, organ morphology, or basic biochemical markers. Such findings are frequently interpreted as evidence of favorable biocompatibility. However, these conclusions should be interpreted cautiously.

Many studies rely on short-term exposure and observation periods, often spanning from only days to weeks. Subtle or delayed toxic effects, immune responses, or cumulative organ burden may therefore remain undetected. Additionally, dose selection is frequently driven by imaging or functional performance rather than realistic exposure scenarios, further limiting translational relevance.

Cheng et al. (2023) explored the therapeutic potential of carbon dots (CDs) synthesized from resveratrol, a naturally occurring compound, using a one-pot green method [108]. These resveratrol-based carbon dots (RES-CDs) were characterized by their enhanced water solubility and robust yellow-green fluorescence under UV light. The study aimed to harness the biocompatible and bioactive properties of RES-CDs for biomedical applications, particularly in wound healing. Extensive testing, including UV-Vis spectroscopy, SEM, TEM, FTIR, XRD, and fluorescence spectroscopy, confirmed the structural and functional integrity of RES-CDs. The biocompatibility and efficacy of RES-CDs were tested both in vitro using human umbilical vein endothelial cells (HUVEC) and in vivo in a rat model. In vitro tests demonstrated that RES-CDs significantly promoted cell migration and tube formation compared to resveratrol alone. In vivo, RES-CDs were injected into male SD rats, showing promising results in accelerating wound healing and stimulating angiogenesis, evidenced by increased expression of CD31 and VEGF in treated skin tissues. Histological analysis performed post-injection revealed no adverse effects in major organs. This study highlights the potential of RES-CDs as a superior alternative to conventional resveratrol in promoting tissue repair and regeneration, opening avenues for their application in clinical settings.

### 5.5. Translational Relevance and Predictive Limitations

A key question in carbon dot biosafety research is whether the results obtained in small organisms or short-term rodent studies are sufficiently predictive for human applications. While zebrafish and invertebrate models provide valuable early-stage hazard identification, they cannot substitute for mammalian models when systemic exposure, metabolism, and immune interactions are critical.

Importantly, the absence of observable toxicity in a given animal model should not be equated with universal safety. Differences in administration route, dose normalization, and material formulation must be carefully considered before extrapolating in vivo findings to human contexts.

Zhang et al. (2019) focused on the impact of graphene quantum dots (GQDs) on male mouse reproductive functions and offspring health, a crucial area given the increasing biomedical use of GQDs [109]. The research explored different exposure methods—oral gavage and intravenous injection—and observed no adverse effects on male sexual behavior, reproductive physiology, or the health of the offspring. Male mice exposed to GQDs displayed normal sexual behaviors, and their reproductive organs, such as testes and epididymides, functioned typically, producing healthy sperm and maintaining normal testosterone levels. Additionally, the offspring produced by GQD-exposed males were healthy, with no apparent differences in health or development compared to controls. The study findings are particularly significant, as they suggest GQDs do not impair male fertility or offspring viability even at high doses, indicating low reproductive toxicity. This low toxicity was confirmed through detailed analyses, including sperm quality assessments and histological examination of reproductive organs, which showed no abnormalities. Furthermore, the offspring showed normal developmental milestones and exhibited no significant health issues, underscoring the potential safety of GQDs in clinical and other applications involving long-term exposure. These results provide valuable insights into the biosafety of GQDs, supporting their use in biomedical applications without affecting male reproductive health or the health of subsequent generations.

### 5.6. Model-Specific and Organ-Specific Toxicity Endpoints Beyond Acute Cytotoxicity

Recent studies have also focused on specific toxicological aspects such as neurotoxicity, hematopoietic toxicity, and microcirculatory toxicity. These research efforts are crucial for understanding the broader implications of nanoparticles on health. The freshwater planarian Dugesia japonica was utilized in a study to investigate the neurotoxic effects of carbon quantum dots (CDs) [110]. The planarians were cut and exposed to CDs, assessing the regeneration of their neural structures. The primary observation was the loss of neuronal regeneration ability and death due to head lysis, linked to interference in the Hedgehog (Hh) signaling pathway, highlighting a novel method for assessing neurotoxicity through regenerative inhibition. In another study, the hematopoietic toxicity of CdTe quantum dots was explored using Bombyx mori larvae [111]. The method involved vascular injections of quantum dots and monitoring their impact on the hematopoietic organs and cells over time. Observations included time- and dose-dependent hematopoietic damage, changes in cell populations, and increased apoptosis, providing a comprehensive method to evaluate hematopoietic toxicity in an invertebrate model.

Although this study concerns CdTe quantum dots rather than carbon dots, it illustrates how invertebrate models can reveal the organ-specific toxicity patterns that may not be captured in standard mammalian assays.

Barros et al. (2022) assessed the impact of high doses of graphene quantum dots (GQDs) on the microcirculation system in a healthy animal model [112]. The method included successive applications of GQDs and subsequent evaluation of microcirculatory damage, demonstrating complete destruction after seven days. This observational approach helps us to understand the circulatory risks associated with GQDs, emphasizing the need for thorough preclinical toxicity assessments in the context of biomedical applications. These findings further underscore that toxicity data obtained for GQDs cannot be directly extrapolated to zero-dimensional carbon dots.

Together, these studies highlight that expanding toxicity assessment beyond conventional endpoints and models can reveal previously overlooked biological effects, while also emphasizing the need for careful interpretation when extrapolating across material classes and species.

## 6. Data-Driven and Machine Learning Approaches in Carbon Dot Toxicity Assessment

Together, these studies highlight that expanding toxicity assessment beyond conventional endpoints and models can reveal previously overlooked biological effects while also emphasizing the need for careful interpretation when extrapolating across material classes and species, as well as for integrative analytical approaches capable of handling such complexity.

Table 3 provides information on various precursors and synthesis environments, cell line studies, and viability percentages. For example, the most common methods used are hydrothermal synthesis and microwave-assisted procedures. In many cases, particularly with some calibrated cell lines, high temperatures help to reduce cytotoxicity. Other cell lines exhibited cytotoxicity at greater doses or under certain circumstances (e.g., MCF-7 had 52% viability). Most carbon quantum dots (CDs) were biocompatible at low concentrations, but increasing concentrations in many cells resulted in a growing harmful impact. Toxicity is also affected by doping.

The table also outlines the various precursors, synthesis settings, cell lines employed, and their viability. Many studies have found that increasing CDs concentrations reduces cell viability. For example, CDs produced from garlic peel have minimal cytotoxicity at lower concentrations, but cause considerable cytotoxicity at higher concentrations. Positively charged CDs (e.g., kernel-coated with polyethyleneimine) are substantially more hazardous than neutral or negatively charged CDs, owing to their cellular penetration ability and consequent destruction of internal cell structures. Toxicity has also been documented for CDs derived from specific precursors such as ammonium citrate or spermidine trihydrochloride, which may induce toxic effects by disrupting antioxidant defenses. The toxicity varies depending on the cell line. As a matter of fact, cancer cell lines (such as HeLa) are usually more resistant at low-to-medium doses, but normal fibroblasts or stem cells may be more vulnerable. Some CDs, such as photodegradable CDs, exhibit much higher cytotoxicity when exposed to light during or after manufacturing than when kept in the dark.

### 6.1. Data-Driven Analysis of the Literature Toxicity Data: A Machine Learning-Assisted Perspective

To assist in organizing and interpreting the highly heterogeneous toxicity data reported for carbon dots, a machine learning-assisted analysis was applied to the literature-derived datasets summarized in Table 1 and Table 2. The primary aim of this analysis was not to generate definitive toxicity predictions, but to identify the synthesis- and testing-related factors that most strongly influence reported biological responses across studies.

For data harmonization, reported cell viability values were grouped into two broad outcome categories. Studies reporting cell viability above approximately 70% were classified as exhibiting low acute cytotoxicity, whereas lower viability values were classified as increased cytotoxic response. This binary categorization does not imply a universal toxicity threshold, but serves as a pragmatic approach to enable cross-study comparison of heterogeneous literature data. A range of machine learning algorithms has previously been applied in nanotoxicity research to relate physicochemical descriptors to biological outcomes (Table 3). Among these, ensemble-based methods such as random forest are particularly suitable for the literature-derived datasets, as they can handle mixed categorical and numerical variables, mitigate overfitting in small datasets, and provide feature importance metrics that are readily interpretable in a materials science context. Accordingly, random forest analysis was used here solely as an exploratory tool to assess feature importance rather than predictive performance.

**Figure 9:**
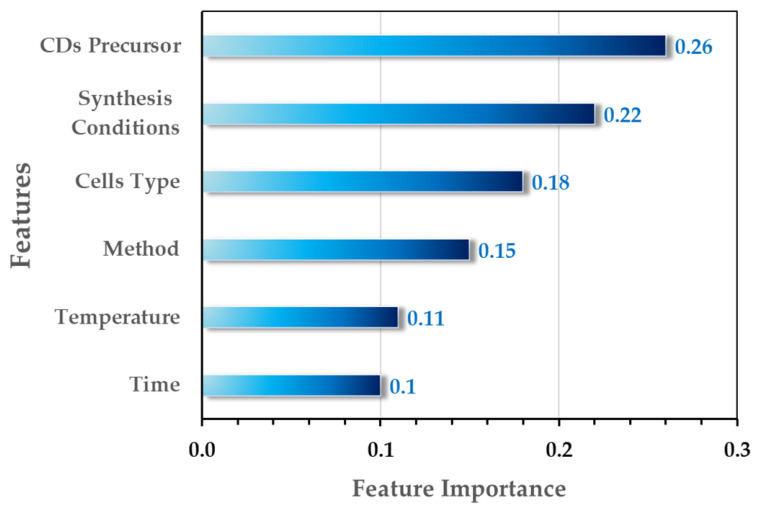
Analysis of the experimental variables associated with toxicity using a random forest model.

As shown in Figure 9, synthesis-related parameters, surface chemistry descriptors, and experimental testing conditions contribute unequally to reported toxicity outcomes. This analysis highlights which variables recur most consistently across the literature as determinants of biological response, complementing the mechanistic discussions presented in earlier sections. Importantly, the limited size and imbalance of the available literature data preclude robust model training or generalizable toxicity prediction. Instead, this data-driven perspective underscores the need for standardized reporting of material properties and toxicity endpoints to enable more reliable integrative analyses in future studies. Among the evaluated approaches, ensemble-based methods such as random forest were found to be particularly suitable for literature-derived nanotoxicity data. This is primarily due to their ability to handle mixed categorical and numerical variables, mitigate overfitting in small datasets, and provide feature importance metrics that are directly interpretable in a materials science context. Rather than focusing on predictive performance, the random forest model was used to explore feature importance across synthesis parameters, surface chemistry descriptors, and testing conditions. This analysis highlights which variables most strongly influence reported toxicity outcomes in the literature-derived dataset, offering a data-driven complement to the mechanistic discussions presented in earlier sections.

### 6.2. Feature Importance Analysis Based on the Literature-Derived Toxicity Data

To explore which parameters most strongly influence reported cytotoxicity outcomes across studies, a feature importance analysis was performed using a random forest model trained on the literature-derived data summarized in Table 1. The input dataset comprised synthesis-related descriptors (e.g., precursor type, synthesis conditions, temperature, reaction time), experimental variables (e.g., cell type, assay method), and corresponding cytotoxicity outcomes reported in the literature.

It is important to emphasize that this analysis does not aim to provide quantitative toxicity prediction. Instead, random forest was employed as an exploratory tool to identify recurring patterns and dominant factors within a heterogeneous and limited dataset. Given the imbalance and relatively small size of available literature data, model performance metrics were not the primary focus.

As shown in Figure 9, precursor type emerges as the most influential feature associated with reported cytotoxicity outcomes, followed by synthesis conditions and cell type. In contrast, individual synthesis parameters, such as reaction temperature and time, exhibit comparatively lower influence. These results are consistent with the mechanistic discussions presented earlier in this review, which emphasize the central role of precursor chemistry in defining surface functionalization, heteroatom incorporation, and downstream biological interactions.

Notably, the prominence of experimental variables alongside material descriptors highlights that reported cytotoxicity is not a solely material-intrinsic property, but also reflects the biological model and testing context. This observation further supports the need for standardized reporting of both synthesis and toxicity assessment parameters to enable more reliable integrative analyses in the future. Figure 9 presents a feature-importance analysis derived from a random forest model trained in the literature-based toxicity data summarized in Table 1 and Table 2. The analysis highlights the relative influence of synthesis-related parameters and experimental conditions on reported cytotoxicity outcomes. Precursor type emerges as the dominant factor, followed by synthesis conditions and cell type, underscoring the primary role of precursor chemistry and experimental context in shaping observed biological responses.

## 7. Photo-Induced Toxicity and Photodegradation of Carbon Dots

### 7.1. Light–Matter Interactions as a Unique Feature of Carbon Dots

Carbon dots are inherently photoactive nanomaterials, with optical properties that underpin many of their most promising applications, including bioimaging, photodynamic therapy, antimicrobial coatings, and photocatalysis. Upon light excitation, CDs can undergo radiative and non-radiative relaxation processes, generate excited charge carriers, and participate in redox reactions. While these features are advantageous for functional applications, they also introduce additional pathways for biological interaction and potential toxicity that are absent under dark conditions.

Consequently, toxicity assessments performed exclusively in the absence of light may underestimate the risks associated with photo-excited carbon dots, particularly in applications involving repeated or prolonged illumination.

In a pioneering study by Miao et al. (2018), the efficacy of sodium humate (SH), derived from humic acids, was explored as a phototheranostic agent for biomedical applications [118]. Humic acids, rich in active groups like phenol, carboxyl, and quinone, have been utilized historically due to their physiological benefits. This study focused on SH’s capabilities in photoacoustic imaging and photothermal therapy, highlighting its superior photothermal conversion efficiency of 76.3%, which outperforms many contemporary photothermal agents. The in vivo applications included successful photoacoustic enhancement and photothermal ablation of HeLa tumors via intratumoral injection. To evaluate in vivo toxicity, mice treated with SH and laser exposure showed no significant pathological changes in major organs (heart, liver, spleen, lung, kidney) or in serum biochemical parameters (ALT, AST, ALP, UA, UREA) compared to a healthy control group. These findings, supported by histological examinations and serum biochemistry assays, underscore the ultralow toxicity and exceptional biocompatibility of SH, marking it as a promising candidate for further development in light-mediated therapeutic and diagnostic applications in biomedicine.

### 7.2. Evidence for Photo-Induced Toxicity

An increasing number of studies have demonstrated that light exposure can significantly alter the biological effects of carbon dots. Under illumination, CDs may generate reactive oxygen species through photo-induced electron–hole separation or energy transfer processes. Elevated ROS levels have been associated with oxidative stress, lipid peroxidation, mitochondrial dysfunction, and cell death in both in vitro and in vivo models.

Importantly, photo-induced toxicity is often reported at CD concentrations that appear non-toxic under dark conditions, highlighting the context-dependent nature of observed biosafety. Differences in excitation wavelength, light intensity, exposure duration, and CD surface chemistry further contribute to variability in reported outcomes.

Numerous publications published over the last 20 years demonstrate the low cytotoxicity (no toxicity) of carbon quantum dots produced from diverse substrates (Table 1 and Figure 9). Interestingly, in 2021, Liu et al. published an article in Nature Communications demonstrating that photodegradation of carbon dots induces cytotoxicity [119]. CDs have been found to be biocompatible in the dark, but they can produce harmful chemicals when exposed to light. Molecular network and community analysis indicate 499 cytotoxicity-related compounds, including 212 with polyethylene glycol, glucose, or benzene-related structures. The authors demonstrated that photo-induced generation of hydroxyl and alkyl radicals plays a key role in the degradation of CDs under varying temperature, pH, light intensity, and wavelength conditions. As a result, future assessments of CDs safety should take into account light exposure or other conditions specific to the application because photodegradation-induced cytotoxicity is likely common to all CDs, regardless of chemical makeup.

### 7.3. Photodegradation and Photo-Responsive Toxicity

Beyond transient photo-induced effects, sustained illumination can lead to the irreversible photodegradation of carbon dots. Photo-oxidative processes may alter surface functional groups, disrupt conjugated domains, and fragment the carbonaceous core, resulting in the formation of low-molecular-weight transformation products. Such degradation products may differ substantially from the parent material in terms of solubility, reactivity, and biological impact. In several studies, photodegraded CDs have been shown to exhibit increased cytotoxicity compared to their pristine counterparts, suggesting that toxicity may arise not only from the nanomaterial itself, but also from its transformation products.

This observation has important implications for long-term exposure scenarios and environmental fate, where CDs may be subjected to prolonged sunlight exposure.

Photo-induced toxicity of carbon dots is particularly relevant in applications where light exposure is intrinsic to their function. In bioimaging and diagnostic imaging, CDs are repeatedly excited with UV or visible light, often in close proximity to sensitive biological structures. Similarly, in photodynamic or antimicrobial applications, ROS generation is deliberately exploited, raising questions regarding off-target effects and material stability.

Environmental exposure scenarios further amplify these concerns. Carbon dots released into surface waters or incorporated into consumer products may undergo continuous solar irradiation, leading to gradual photodegradation and the formation of potentially bioactive byproducts. In such contexts, toxicity assessments conducted under dark laboratory conditions may fail to capture environmentally relevant risks.

The photo-responsive nature of carbon dots underscores the necessity of incorporating light conditions into toxicity evaluation protocols. Studies that omit illumination parameters may provide an incomplete or misleading picture of biosafety, particularly for photoactive applications. The key considerations include reporting excitation wavelength, light intensity, exposure duration, and whether toxicity endpoints are evaluated under illuminated or dark conditions. Without such information, comparison across studies and risk assessment remain challenging.

## 8. Biodegradation and Environmental Toxicity of Carbon Dots

### 8.1. Distinguishing Biodegradation from Physicochemical Transformation

In the context of carbon dot biosafety, the term “biodegradation” is often used ambiguously and may encompass a range of processes, including enzymatic breakdown, photo-oxidative transformation, hydrolysis, and surface chemical modification. Unlike conventional biodegradable polymers, carbon dots do not undergo a single, well-defined degradation pathway. Instead, their transformation in biological and environmental systems is governed by a combination of physicochemical and biological factors. It is therefore important to distinguish true biodegradation—involving biological processing and molecular breakdown—from abiotic transformations such as photodegradation or oxidative surface modification. Both processes can alter the identity and biological behavior of CDs, but they may result in distinct transformation products with different toxicological implications.

### 8.2. Factors Influencing Degradation and Persistence

The susceptibility of carbon dots to degradation is strongly dependent on their size, surface chemistry, degree of graphitization, and functionalization. CDs with highly oxidized or defect-rich surfaces are generally more prone to chemical and photo-induced transformations, whereas materials with higher structural order may exhibit greater persistence. Environmental conditions further modulate these processes. Exposure to sunlight, oxygen, variable pH, and natural organic matter can accelerate surface oxidation and fragmentation. In biological environments, interactions with enzymes, reactive oxygen species, and cellular metabolites may contribute to gradual chemical modification, even in the absence of complete mineralization.

These factors suggest that degradation behavior is highly context-specific and cannot be inferred solely from precursor type or synthesis method. It is worth noting that degradation products may have different chemical properties and toxicity profiles.

### 8.3. Environmental Fate and Aquatic Toxicity

Environmental release of carbon dots may occur during synthesis, application, disposal, or through degradation of CD-containing products. Once released into aquatic systems, CDs can interact with microorganisms, algae, invertebrates, and fish, making aquatic toxicity a key component of environmental risk assessment.

Studies examining the effects of CDs on algae, Daphnia, and zebrafish embryos have reported a wide range of outcomes, from negligible effects to growth inhibition, oxidative stress, and developmental abnormalities. As with biomedical toxicity studies, these discrepancies are often linked to differences in CD properties, exposure concentrations, and experimental design.

Importantly, photodegraded or environmentally transformed CDs may exhibit toxicity profiles distinct from those of pristine materials. This observation underscores the need to evaluate not only the parent nanomaterial, but also its potential transformation products when assessing environmental impact.

### 8.4. Challenges in Assessing Environmental Toxicity

A major challenge in evaluating the environmental toxicity of carbon dots lies in the lack of standardized testing protocols. Exposure concentrations used in laboratory studies frequently exceed environmentally relevant levels, while short-term assays may fail to capture chronic or sublethal effects. Furthermore, the intrinsic fluorescence of CDs can interfere with common ecotoxicological endpoints, complicating the interpretation of the results. Limited attention has also been paid to the role of aggregation, sedimentation, and interaction with natural organic matter, all of which can influence bioavailability and toxicity in environmental systems.

### 8.5. Implications for Risk Assessment and Sustainable Design

The potential for environmental persistence, transformation, and ecotoxicity highlights the importance of integrating degradation behavior into the safety evaluation of carbon dots. Materials designed for biomedical or consumer applications may ultimately enter environmental compartments, where exposure scenarios differ markedly from those considered in laboratory studies.

In this context, the frequent use of natural or biomass-derived precursors should not be equated with inherent environmental safety. While such approaches may reduce synthesis-related hazards, the environmental fate of the resulting CDs depends on their final physicochemical properties rather than their origin.

## 9. Reconciling Reactive Oxygen Species Generation and Antioxidant Behavior of Carbon Dots

### 9.1. Apparent Contradictions in Reported Redox Activity

One of the most frequently reported yet seemingly contradictory observations in the literature on carbon dots concerns their redox behavior. Numerous studies describe carbon dots as inducers of reactive oxygen species (ROS), linking elevated intracellular ROS levels to oxidative stress, mitochondrial dysfunction, and cytotoxicity. In contrast, other reports attribute antioxidant or ROS-scavenging properties to CDs, suggesting protective effects against oxidative damage.

At first glance, these findings appear mutually exclusive. However, a closer examination reveals that ROS generation and antioxidant behavior are not intrinsic, fixed properties of carbon dots, but rather context-dependent outcomes governed by material characteristics and experimental conditions.

In their innovative study, Tang et al. developed a metal-free heterojunction photocatalyst composed of carbon quantum dots (CDs) integrated with graphitic carbon nitride (g-C3N4), aimed at combating antibiotic-resistant strains of Staphylococcus aureus [120]. The newly synthesized CDs/g-C3N4 demonstrated enhanced photocatalytic inactivation of *S. aureus* under visible light, notably outperforming pure g-C3N4. This point was attributed to a significant increase in intracellular reactive oxygen species and subsequent disruption of bacterial cell membranes, leading to bacterial death. The effectiveness of CDs/g-C3N4 was further validated through a cutaneous infection model in mice, where it substantially reduced bacterial loads and facilitated lesion recovery. In vivo and in vitro toxicity assessments revealed negligible side effects, with no notable difference in lesion area among different groups and no impact on HaCaT cell viability at concentrations up to 10 mg/mL. These findings underscore the potential of CDs/g-C3N4 as a safe and effective photocatalyst for treating *S. aureus* infections, offering a promising alternative to traditional antimicrobial strategies that often lead to resistance.

### 9.2. Influence of Surface Chemistry and Doping on Redox Behavior

Surface functional groups play a central role in determining the redox activity of carbon dots. Oxygen-containing functionalities, surface defects, and heteroatom dopants such as nitrogen can introduce redox-active sites that either promote or quench ROS, depending on their chemical environment and electronic structure.

Nitrogen-doped CDs, for example, are frequently reported to exhibit enhanced electron-donating or electron-accepting behavior, which may facilitate ROS generation under photoexcitation while enabling antioxidant activity under dark or low-stress conditions. As a result, identical materials may appear pro-oxidant or antioxidant depending on illumination, oxygen availability, and cellular redox state.

### 9.3. Dose-Dependent and Non-Linear Responses

Dose represents another critical determinant of observed redox behavior. Several studies suggest non-linear or biphasic dose–response relationships, in which low concentrations of CDs exert antioxidant or cytoprotective effects, while higher concentrations induce oxidative stress and cytotoxicity. Such hormetic responses are well documented for other nanomaterials and redox-active compounds. Unfortunately, due to the chemical complexity of CQDs, their properties cannot be compared to other nanomaterials such as silver or gold nanoparticles [121] or CdS quantum dots [122]. This means that the knowledge base regarding their toxicity is almost built from scratch.

Failure to account for these nonlinear effects, especially when drawing conclusions from single-dose trials, might result in simplistic and sometimes misleading interpretations of CD biosafety.

### 9.4. Role of Experimental Context and Assay Limitations

Experimental conditions strongly influence ROS-related observations. Illumination parameters, exposure duration, and the presence of serum proteins or antioxidants in culture media can all modulate redox responses. Moreover, commonly used ROS detection assays are susceptible to interference from the intrinsic fluorescence and redox activity of carbon dots themselves. These methodological limitations further complicate interpretation and may contribute to apparent discrepancies between studies. Without appropriate controls and validation, distinguishing genuine biological ROS generation from assay artifacts remains challenging.

### 9.5. A Unified Conceptual Framework for Interpreting Redox Effects

Rather than viewing ROS generation and antioxidant behavior as contradictory phenomena, they should be understood as different manifestations of the same underlying material properties operating under distinct conditions. Carbon dots occupy a continuum of redox activity defined by surface chemistry, doping, concentration, and environmental context. Within this framework, CDs may act as redox buffers at low concentrations or under mild conditions, while transitioning to ROS-generating agents under higher stress, photoexcitation, or elevated doses. This perspective reconciles divergent reports in the literature and emphasizes the importance of context-aware interpretation.

## 10. Toward Standardized Toxicity Evaluation Protocols for Carbon Dots

### 10.1. The Need for Standardization in Carbon Dot Toxicity Studies

The wide variability in reported toxicity outcomes for carbon dots not only reflects material heterogeneity, but also substantial inconsistencies in experimental design, characterization depth, and data reporting. At present, there is no widely accepted framework for evaluating CD biosafety, resulting in studies that are difficult to compare and, in some cases, challenging to interpret. Without standardized assessment strategies, the accumulation of toxicity data risks reinforcing fragmentation rather than advancing understanding. Establishing minimal yet practical guidelines for toxicity evaluation and reporting is therefore essential for improving reproducibility, enabling cross-study comparison, and supporting translational progress.

In many studies, the in vivo toxicity evaluation of carbon quantum dots (CDs) on animals employs a variety of methods, including histological examinations, hematological, and biochemical analyses. These approaches provide insight into the effects of CDs on major organs such as the heart, liver, spleen, lungs, and kidneys. However, not all studies systematically conduct these evaluations, and some assessments may be overly simplistic, leading to conclusions about the safety of CDs that might not be fully convincing. A systematic approach to evaluating CQD toxicity in vivo should include a comprehensive set of tests: histopathological analysis for organ damage, hematological profiles to check for blood cell viability, biochemical markers to assess liver and kidney function, and some specific assays to measure any neurological and reproductive impacts, ensuring a thorough safety assessment.

As for the differences in toxicity evaluation based on the route of administration, studies involving oral administration of CDs reveal additional considerations compared to other methods such as intravenous or intraperitoneal injections. In addition to assessing damage to vital organs, it is crucial to evaluate the impact on gastrointestinal mucosal absorption, the gut microbiome, and liver functions (potentially involving first-pass effects). Oral administration studies must carefully consider how CDs interact with the digestive system, from absorption to potential effects on intestinal health and microbial balance, which are not typically concerns with other administration routes. These factors make oral toxicity studies particularly complex and critical for understanding the full impact of CDs when administered in this manner.

### 10.2. Minimal Physicochemical Characterization Requirements

Reliable toxicity assessment must be grounded in adequate physicochemical characterization of the tested material. At a minimum, studies should report core size and morphology (e.g., TEM or AFM), hydrodynamic size (DLS) and aggregation behavior in relevant exposure media, surface charge (zeta potential), and key surface functional groups. XPS and FT-IR are the key analytical techniques for investigating the surface functional groups of carbon quantum dots. Under these conditions, both methods are highly complementary: XPS provides detailed information on elemental composition and chemical states at the surface, while FT-IR offers insights into the vibrational signatures of functional groups. Consequently, they are very often used together to obtain a comprehensive and reliable characterization of CQD surface chemistry.

Given the sensitivity of biological responses to subtle surface variations, characterization should ideally be performed not only in water, but also in biologically relevant media. When heteroatom doping or post-synthetic functionalization is employed, its extent and chemical nature should be clearly documented. In practice, techniques such as XPS are not always optimal for studying surface functional groups, as they require solid, dried samples whose surface chemistry may change during sample preparation. These preparations induced alterations can obscure the true chemical state of carbon dots in their native, colloidal form. For this reason, ATR FT-IR often represents a more suitable analytical approach, as it allows for the characterization of samples in hydrated or minimally altered states. However, the strong absorption band of water may complicate spectral interpretation, particularly in the fingerprint region, and must be carefully accounted for during analysis.

### 10.3. Recommended In Vitro Toxicity Assessment Framework

In vitro toxicity testing carbon dots should move beyond single-endpoint screening toward a multiparametric approach. Core endpoints should include cell viability, membrane integrity, oxidative stress, and indicators of apoptosis or necrosis, assessed across a range of concentrations and exposure durations.

Importantly, studies should explicitly evaluate potential assay interference arising from the optical and redox properties of CDs. Appropriate controls—including particle-only, assay-only, and medium-only conditions—are necessary to distinguish genuine biological effects from methodological artifacts.

### 10.4. Integration of In Vivo Studies and Exposure Relevance

For applications involving systemic exposure, in vivo toxicity studies remain indispensable. Such studies should be designed with careful consideration of dose selection, administration route, and observation period. Short-term acute toxicity assessments, while informative, should not be interpreted as definitive evidence of long-term safety.

Reporting of biodistribution, clearance pathways, and organ-specific accumulation is particularly important for contextualizing observed toxicity outcomes. Where feasible, integration of in vivo findings with in vitro mechanistic data can enhance interpretability and predictive value.

### 10.5. Reporting, Reproducibility, and Batch Considerations

Given the inherent synthesis-dependent variability of carbon dots, toxicity studies should report batch identifiers and, where possible, include characterization of multiple batches. Even limited assessment of inter-batch variability can substantially strengthen the robustness of reported conclusions.

Transparent reporting of synthesis protocols, purification steps, and characterization methods is critical for reproducibility. Adoption of standardized reporting checklists—analogous to emerging practices for other nanomaterials—would represent a significant step forward for the field.

### 10.6. Role of Data-Driven Approaches in Supporting Standardization

The growing volume of toxicity data on carbon dots presents both challenges and opportunities. Data-driven approaches, including machine learning, offer promising tools for identifying governing trends across heterogeneous datasets, provided that input data are sufficiently standardized and well annotated.

Rather than replacing experimental studies, such approaches can complement traditional toxicity assessment by highlighting key determinants of biological response and guiding rational material design. Their successful implementation, however, is contingent on improved data quality, consistency, and transparency.

## 11. Batch-to-Batch Variability and Reproducibility Challenges in Carbon Dot Research

### 11.1. Origins of Batch-to-Batch Variability

Batch-to-batch variability is an intrinsic challenge in carbon dot research, arising primarily from the bottom-up nature of most synthesis approaches. Small fluctuations in precursor composition, purity, reaction temperature, heating rate, or post-synthetic purification can lead to measurable differences in particle size distribution, surface functionalization, heteroatom content, and residual molecular species.

Unlike well-defined molecular compounds or highly crystalline inorganic nanomaterials, carbon dots represent heterogeneous ensembles rather than single, uniform entities. As a consequence, even minor deviations in synthesis conditions may result in batches with distinct physicochemical and biological profiles, despite being nominally prepared using identical protocols.

### 11.2. Impact on Toxicity Assessment and Data Interpretation

From a toxicological perspective, batch-to-batch variability can significantly influence reported outcomes. Biological systems often respond sensitively to changes in surface chemistry, charge distribution, and redox activity, all of which may vary between batches. As a result, differences in cytotoxicity, oxidative stress induction, or biodistribution observed across studies may reflect material variability rather than true contradictions in biological behavior.

Importantly, many toxicity studies evaluate only a single batch of carbon dots and implicitly assume reproducibility. Such practices limit the reliability of safety conclusions and complicate cross-study comparisons, particularly when subtle biological effects are under investigation.

### 11.3. Current Reporting Gaps and Reproducibility Limitations

Despite the recognized importance of reproducibility, explicit discussion of batch-to-batch variability remains rare in the carbon dot literature. In many cases, synthesis protocols are described qualitatively, without sufficient detail to enable independent replication. Batch identifiers, yield variability, and inter-batch characterization data are seldom reported.

This lack of transparency hampers reproducibility and undermines confidence in reported toxicity data. Moreover, it poses a significant obstacle to the development of data-driven approaches, which rely on consistent and well-annotated datasets to extract meaningful patterns.

The choice of heating modality significantly dictates the final morphology and properties of the nanomaterial, as evidenced by studies comparing hydrothermal and microwave-assisted syntheses. For instance, CQDs derived from the same precursors under optimized conditions exhibited distinct average diameters: 4.52 nm for the hydrothermal method versus 6.08 nm for the microwave method [123]. This discrepancy highlights that reporting the process temperature alone is insufficient for ensuring reproducibility. Microwave irradiation facilitates rapid, volumetric heating through direct molecular interaction, leading to nearly instantaneous and uniform thermal distribution throughout the reaction mixture. In contrast, conventional hydrothermal heating relies on slower, conductive heat transfer from the vessel walls, creating thermal gradients. Such variations in heating rates (ramp-up times) fundamentally influence the kinetics of nucleation and subsequent grain growth, thereby altering the final particle size, surface oxidation states, and overall photoluminescence profiles.

### 11.4. Practical Recommendations for Addressing Batch Variability

To improve reproducibility and interpretability, future studies should explicitly acknowledge and assess batch-to-batch variability. At a minimum, toxicity evaluations should report whether results were obtained from a single batch or multiple independently synthesized batches. Where feasible, basic physicochemical characterization should be performed for each batch tested.

Establishing internal reference materials or standardized synthesis benchmarks within individual laboratories may further enhance consistency. Over time, community-wide adoption of such practices could support inter-laboratory comparison and contribute to the development of more reliable safety assessment frameworks.

The synthesis of CQDs is highly sensitive to reaction conditions, precursor materials, and synthesis methods, all of which impact particle size distribution and photoluminescence properties. Addressing these factors through standardized protocols and optimization techniques is essential for improving reproducibility and reducing batch-to-batch variability in CQDs synthesis.

The high sensitivity of the synthesis process to experimental parameters carries a significant risk that observed results may lack reproducibility. Researchers frequently base their conclusions on the examination of a single production batch, citing average diameter and size dispersion, instead of performing multi-batch validation. However, in the realm of quantum-confined materials, a discrepancy as small as 1 nm is critically significant; such a marginal shift can fundamentally alter photoluminescence spectra, surface chemistry, and, consequently, the material’s toxicological profile.

## 12. Conclusions and Future Perspectives

Carbon dots have rapidly evolved from a laboratory curiosity into a widely explored class of carbon-based nanomaterials with growing relevance for biomedical, environmental, and technological applications. Despite this progress, the present review highlights that the current understanding of carbon dot cytotoxicity and biosafety remains fragmented and strongly context dependent. Apparent contradictions in reported toxicity outcomes do not reflect the intrinsic inconsistencies of the material itself, but rather arise from synthesis-dependent heterogeneity, methodological limitations, and insufficiently standardized evaluation strategies.

A central conclusion of this review is that carbon dot toxicity cannot be meaningfully discussed in binary terms such as “toxic” or “non-toxic.” Instead, biological responses emerge from the interplay between physicochemical properties, synthesis history, exposure conditions, and experimental context. Factors such as surface chemistry, heteroatom doping, dose, illumination, and material transformation over time jointly define biological outcomes and must be considered in an integrated manner.

Importantly, statistical analysis of the compiled dataset indicates that the apparent low toxicity of carbon dots reported in the literature may partly reflect the dominance of specific cellular models, particularly HeLa cells, which are frequently used in toxicity assays. When toxicity outcomes are analyzed across different biological models, substantial variability emerges, suggesting that the biological context plays a critical role in determining observed cytotoxic responses.

Looking forward, several key challenges must be addressed to advance the field toward reliable safety assessment and responsible application. First, greater emphasis should be placed on synthesis reproducibility and batch-to-batch variability, with explicit reporting of material identity and inter-batch differences. Without such transparency, the accumulation of toxicity data risks reinforcing ambiguity rather than resolving it. Second, toxicity evaluation strategies should evolve from single-endpoint screening toward multiparametric and context-aware frameworks that account for assay interference, non-linear dose responses, and photo-induced effects.

Environmental fate and transformation represent another critical frontier. As carbon dots increasingly enter consumer products and biomedical applications, understanding their long-term behavior under realistic environmental and biological conditions—including photodegradation and the formation of transformation products—will be essential for comprehensive risk assessment. The frequent use of natural or biomass-derived precursors, while attractive from a sustainability perspective, should not be equated with inherent safety without rigorous evaluation of the resulting materials.

Importantly, the growing volume of published data also presents an opportunity. Data-driven approaches, including machine learning, hold promise for identifying governing trends across heterogeneous datasets and guiding the rational design of safer carbon dots. However, the successful application of such tools is contingent upon improved data quality, standardized reporting, and careful annotation of experimental context. In this sense, advances in computational analysis and experimental standardization must proceed hand in hand.

The future of carbon dot research lies not in further expanding the number of isolated toxicity reports, but in consolidating existing knowledge through critical comparison, methodological rigor, and integrative analysis. By adopting standardized evaluation protocols and embracing context-aware interpretation, the field can move toward a more predictive, reproducible, and application-relevant understanding of carbon dot biosafety.

Taken together, the reviewed studies indicate that variability in carbon dot synthesis—encompassing precursor selection, reaction conditions, and post-synthesis modification—remains a primary source of inconsistency in reported physicochemical properties and biological responses. Rather than isolated parameters, it is the coupled influence of size, surface chemistry, charge, and aggregation behavior that ultimately governs toxicity outcomes. Addressing this complexity will require not only improved experimental standardization, but also integrative analytical approaches capable of reconciling heterogeneous literature data.

## Figures and Tables

**Table 3:** Machine learning (ML) algorithms are commonly applied in nanotoxicity studies and their typical use cases.

ML Algorithm	Advantages	Disadvantages	Use Case in Nanomaterials Toxicity
Logistic Regression	Logistic regression is suitable for classification and can handle both categorical and numerical data and is easy to implement.	Logistic regression is not efficient when the data is complex, and can assume linearity between independent and dependent variables.	Logistic regression has already found many applications in finding the toxicity of nanomaterials by analyzing the biological interaction and physicochemical properties [113].
Support Vector Machines (SVM)	SVM is good for linear and nonlinear data, and it is good for overcoming overfitting in high-dimensional data	SVM is a complex and computationally intensive algorithm that is difficult to interpret. It requires careful selection of the kernel.	Researchers used SVM to classify the nanomaterials based on toxicity by observing their physicochemical properties and resistance to bacteria [114,115].
K-Nearest Neighbors (KNN)	KNN is a straightforward and simple method. It can handle multi-class data. KNN is good for small- to medium-sized data.	KNN becomes complex and intensive for large datasets. It can struggle for high-dimensional data. It requires careful selection of distance of metrics.	KNN was used by researcher to examine their biological properties and determine their toxicity [114,115].
Decision Trees (DT)	DT can also handle both categorical and numerical data well, and they are easy to understand and require little data processing.	Decision trees can be biased if the data are not appropriate for each class or material type Not good for very complex data.	Decision trees were used by researchers to examine biological interactions and determine toxicity [116].
Random Forest	It can handle high-dimensional data and reduce overfitting by using an averaging function, and provide feature importance graphs, which are essential for material selection.	Random forest can become slow for real-time data and challenging to interpretas compared to a single decision tree.	Random forest is widely used in the selection of nanomaterials because it supports feature importance analysis and robust classification evaluation [114,117].

## Data Availability

The data analyzed in this study are derived from previously published articles cited throughout the manuscript. No new experimental data were generated. The processed dataset used for the statistical and machine learning analyses was compiled from the literature sources listed in Table 1 and Table 2. The curated dataset supporting the conclusions of this article is available from the corresponding author upon reasonable request.
